# Biogeographic survey of soil bacterial communities across Antarctica

**DOI:** 10.1186/s40168-023-01719-3

**Published:** 2024-01-12

**Authors:** Gilda Varliero, Pedro H. Lebre, Byron Adams, Steven L. Chown, Peter Convey, Paul G. Dennis, Dandan Fan, Belinda Ferrari, Beat Frey, Ian D. Hogg, David W. Hopkins, Weidong Kong, Thulani Makhalanyane, Gwynneth Matcher, Kevin K. Newsham, Mark I. Stevens, Katherine V. Weigh, Don A. Cowan

**Affiliations:** 1 Department of Biochemistry, Genetics and Microbiology, Centre for Microbial Ecology and Genomics, University of Pretoria, Pretoria, 0002 South Africa; 2 Rhizosphere Processes Group, Swiss Federal Research Institute WSL, 8903 Birmensdorf, Switzerland; 3 Department of Biology, Brigham Young University, Provo, UT 84602 USA; 4 Monte L. Bean Life Science Museum, Brigham Young University, Provo, UT 84602 USA; 5 Securing Antarctica’s Environmental Future, School of Biological Sciences, Monash University, Clayton, VA 3800 Australia; 6 British Antarctic Survey, Natural Environment Research Council, High Cross, Madingley Road, Cambridge, CB3 0ET UK; 7 Department of Zoology, University of Johannesburg, PO Box 524, Auckland Park, 2006 South Africa; 8 Biodiversity of Antarctic and Sub-Antarctic Ecosystems (BASE), Santiago, Chile; 9 School of the Environment, The University of Queensland, Brisbane, QLD 4072 Australia; 10 State Key Laboratory of Tibetan Plateau Earth System, Environment and Resources (TPESER), Institute of Tibetan Plateau Research, Chinese Academy of Sciences, Beijing, 100101 China; 11 School of Biotechnology and Biomolecular Sciences, University of NSW, Sydney, NSW 2052 Australia; 12 School of Science, University of Waikato, Hamilton, New Zealand; 13 Canadian High Arctic Research Station, Polar Knowledge Canada, Cambridge Bay, NU Canada; 14 SRUC - Scotland’s Rural College, West Mains Road, Edinburgh, EH9 3JG Scotland, UK; 15 Department of Biochemistry, Genetics and Microbiology, University of Pretoria, Pretoria, 0002 South Africa; 16 Department of Biochemistry and Microbiology, Rhodes University, Makhanda, South Africa; 17 Securing Antarctica’s Environmental Future, Earth and Biological Sciences, South Australian Museum, Adelaide, SA 5000 Australia; 18 School of Biological Sciences, University of Adelaide, Adelaide, SA 5005 Australia

**Keywords:** Antarctic Conservation Biogeographic Regions (ACBRs), Antarctic soil microbiome, Biogeography, Microbial diversity, Regionalization, Soils, Bioclimatic variables

## Abstract

**Background:**

Antarctica and its unique biodiversity are increasingly at risk from the effects of global climate change and other human influences. A significant recent element underpinning strategies for Antarctic conservation has been the development of a system of Antarctic Conservation Biogeographic Regions (ACBRs). The datasets supporting this classification are, however, dominated by eukaryotic taxa, with contributions from the bacterial domain restricted to Actinomycetota and Cyanobacteriota. Nevertheless, the ice-free areas of the Antarctic continent and the sub-Antarctic islands are dominated in terms of diversity by bacteria. Our study aims to generate a comprehensive phylogenetic dataset of Antarctic bacteria with wide geographical coverage on the continent and sub-Antarctic islands, to investigate whether bacterial diversity and distribution is reflected in the current ACBRs.

**Results:**

Soil bacterial diversity and community composition did not fully conform with the ACBR classification. Although 19% of the variability was explained by this classification, the largest differences in bacterial community composition were between the broader continental and maritime Antarctic regions, where a degree of structural overlapping within continental and maritime bacterial communities was apparent, not fully reflecting the division into separate ACBRs. Strong divergence in soil bacterial community composition was also apparent between the Antarctic/sub-Antarctic islands and the Antarctic mainland. Bacterial communities were partially shaped by bioclimatic conditions, with 28% of dominant genera showing habitat preferences connected to at least one of the bioclimatic variables included in our analyses. These genera were also reported as indicator taxa for the ACBRs.

**Conclusions:**

Overall, our data indicate that the current ACBR subdivision of the Antarctic continent does not fully reflect bacterial distribution and diversity in Antarctica. We observed considerable overlap in the structure of soil bacterial communities within the maritime Antarctic region and within the continental Antarctic region. Our results also suggest that bacterial communities might be impacted by regional climatic and other environmental changes. The dataset developed in this study provides a comprehensive baseline that will provide a valuable tool for biodiversity conservation efforts on the continent. Further studies are clearly required, and we emphasize the need for more extensive campaigns to systematically sample and characterize Antarctic and sub-Antarctic soil microbial communities.

Video Abstract

**Supplementary Information:**

The online version contains supplementary material available at 10.1186/s40168-023-01719-3.

## Introduction

Antarctica is the coldest, windiest, and driest continent, posing some of the harshest and most challenging conditions for life [1]. The vast majority of the continent is permanently covered by ice, with less than 0.3% of its continental area being ice-free [2]. However, these areas include a wide range of geographical/geological features and a total area of over 20,000 km^2^ [2–4]. Ice-free areas are typically small exposed islands of land surrounded by ice or ocean, with those hosting most biodiversity mainly located in proximity to the coast along the Antarctic Peninsula and its associated Scotia Arc archipelagoes, and in coastal oases around the edge of the Antarctic continent. They also include the hyperarid deserts of the Victoria Land Dry Valleys (individually the largest ice-free areas in Antarctica), large inland mountain ranges such as the Transantarctic Mountains, and Ellsworth Mountains, and smaller ranges and isolated inland nunataks [5–7]. These systems are generally characterized by exposure to a combination of extreme environmental stresses, including high UV radiation, low water availability, high salinity, low temperatures, and low nutrient availability, posing multiple challenges to life [8].

The Antarctic continent is commonly divided into maritime and continental zones in descriptions of its biodiversity [6, 9, 10]. Under this definition, maritime Antarctica comprises the western coastal regions and offshore islands of the Antarctic Peninsula and the Scotia Arc archipelagoes of the South Shetland, South Orkney, and South Sandwich Islands, plus the isolated oceanic islands of Bouvet and Peter I [11]. The much larger area of continental Antarctica comprises the geological regions of East and West Antarctica and their offshore islands, plus the eastern coastal regions of the Antarctic Peninsula. However, the latter was historically included under the “continental Antarctica” definition primarily due to the comparability of its climatic conditions, as virtually no biological survey and diversity data exist for the eastern Peninsula. Beyond the maritime and continental Antarctic regions, the sub-Antarctic region lies at mid-latitudes in the Southern Ocean and consists of a range of isolated islands generally in proximity to the Antarctic Polar Front [6, 12]. Beyond the “core” sub-Antarctic, further peri-Antarctic islands are recognized at lower latitudes, whose biodiversity overlaps with that of the formally defined sub-Antarctic Islands [12]. The maritime Antarctica is characterized by slightly higher summer temperatures and greater precipitation compared to continental Antarctica, while the sub-Antarctic islands are generally cool with only limited seasonal variation in environmental conditions and a considerable proportion of precipitation as rain [13, 14]. In the continental Antarctica, precipitation occurs almost entirely in the form of snow and is often extremely limited. In some parts of the continent where low atmospheric humidity is typical, much of this snow undergoes sublimation and is lost from terrestrial ecosystems before wetting of the underlying soil can occur. Common features of continental Antarctic soils are low moisture and nutrient concentrations, subzero temperatures for an extended period of each annual cycle, and frequent soil freeze–thaw cycles during the austral summer [10, 15]. Antarctic soils are also characterized by different formation histories and environmental factors (e.g., humidity, temperature, UV radiation, salinity, pH and carbon, and nutrient availability) [4, 8, 10, 16–19].

These diverse climate characteristics and soil properties influence macro- and micro-biological diversity patterns [14, 20, 21]. The harsh environmental conditions mean that the continent hosts only two native species of vascular plants, present exclusively on the Antarctic Peninsula and maritime Antarctic archipelagoes, where there is a much higher diversity and wider distribution of bryophytes and lichens compared to vascular plants [6, 22–24]. Marine vertebrate breeding colonies, and haul-out and molting areas, are present around the continent, with the vast majority in close proximity to the coast, where soil nutrients are locally enriched in marine-derived nutrients introduced by animal activity [25–27]. Invertebrate groups, such as springtails, mites, tardigrades, rotifers and nematodes, are often common but with varying patterns of species distributions across the continent [21, 28–32]. Some groups that are otherwise widely regarded to be cosmopolitan, such as nematodes, appear to be completely absent in certain regions even though other micro-invertebrates are present [33, 34], and many show high degrees of species-level endemism at various spatial scales [21, 32, 35–37]. Compared to plants and animals, microorganisms (e.g., bacteria, archaea, fungi) are numerically and phylogenetically dominant across the Antarctic continent [8, 38–41].

The unique biodiversity hosted by this largely pristine continent is currently threatened by a number of factors [42], including climate change where new climatic conditions could destabilize biotic equilibria [43], the introduction of new non-native species [44, 45], and direct physical human impacts [46–48]. The unique and largely pristine nature of terrestrial Antarctica (south of 60°) has been subject to Antarctic Treaty governance since 1961 and is currently protected under the Protocol on Environmental Protection to the Antarctic Treaty, whose requirements have led to several international research initiatives focusing on the current and future protection and conservation of the region [49–51]. Drawing on this initiative, the cataloged distribution patterns of micro- and macro-invertebrates, plants, algae, and some microorganisms have been used to define a series of Antarctic Conservation Biogeographic Regions (ACBRs) [52, 53]. The current definition of 16 ACBRs was based on the distribution of 1823 taxa derived from 38,854 biological records and expert-defined bioregions. However, it was also recognized that there was uneven distribution of data across both taxa and regionally within Antarctica. The available data represented vascular and non-vascular plants (4 and 258 taxa, respectively), metazoa (153 taxa), multi-cellular algae (182 taxa), eukaryotic microalgae and protists (283 taxa), fungi (760 taxa), and bacteria (183 taxa). It is notable that only 10% of the available taxa and 3% of the records belonged to the bacterial domain, and only to the phyla Actinomycetota and Cyanobacteriota [52]. A good fit into the ACBR frame has been reported for diatoms in Antarctic lakes [54], but no previous studies have been reported that test the distribution of much broader taxonomic groups (i.e., total microbiomes) within the context of the ACRB framework. Further, to date, no studies analyzing Antarctic bacterial community patterns at a continental scale have been conducted using data generated by high-throughput sequencing technology, despite the general upsurge in the application of these modern molecular biological approaches.

Regional and local biogeographic surveys suggest that prokaryotic distribution patterns are complex, varying in relation to the studied area and scale, driven by both abiotic and biotic factors, and potentially impacted by different dispersal mechanisms [7, 14, 20, 39, 55–59]. High soil microbial community spatial heterogeneity, arising from variability in edaphic factors, has been observed in some Antarctic areas such as the continental Antarctic Ross Sea region [39, 55, 60, 61] and maritime Antarctic Signy Island [62]. Some prokaryotic taxa are potentially endemic to the Antarctic continent or specific Antarctic areas [43, 63–66], while others show cosmopolitan distributions, possibly indicative of different past and ongoing dispersal strategies [20, 67]. Despite the existence of multiple prokaryote phylogenetic datasets from many Antarctic ice-free areas, very few of these data have yet contributed to the development of the ACBR classifications.

In this study, we set out to conduct the first continental-scale biogeographic survey of soil bacterial communities across Antarctica. We used 16S rRNA gene amplicon datasets to investigate bacterial phylogenetic patterns at a continental scale (i.e., distance-decay analysis) and assess major environmental drivers likely to contribute to these patterns (e.g., temperature, precipitation). Our aim was to assess whether any bacterial distribution patterns identified were consistent with and/or could provide a valuable addition to the current ACBR classification system. Furthermore, we compared how bacterial communities from mainland and island samples differed and investigated how these communities were shaped by bioclimatic variables such as air temperature and precipitation.

## Materials and methods

### Dataset creation

Our dataset comprised 1164 samples from 17 16S rRNA gene sequencing datasets (Table S1) derived from shallow (0–10 cm depth) soils. Soil storage conditions and DNA extraction methods are reported in Table S1. These 17 datasets were all derived from the 16S rRNA gene but spanned five distinct 16S rRNA regions, viz*.*, V1–V3, V3–V4, V4, V4–V5, and V8–V9. The primer pairs used for amplification were 27F–519R and pA–BKL1 for V1–V3, 341F–805R and 341F–806R for V3–V4, 515F–806R for V4, 515F–926R for V4–V5, and 926F–1392wR for V8–V9 (Table S1). All datasets were collated from online repositories and collaborators except for dataset 10, which was obtained by resequencing samples collected from the Prince Charles Mountains and coastal areas in Eastern Antarctica ACBR [68–70]. 16S rDNA amplicon libraries were prepared using the KAPA HiFi PCR kit (Roche) and sequenced using Illumina MiSeq technology (paired-end, 300 cycles) by Omega Bioservices (Norcross, USA). Data availability for all datasets is reported in Table S1.

### Bioclimatic variables and metadata

Bioclimatic variables (1981–2010) were extracted from the CHELSA database v 2.1 [71] using the R package terra v 1.6–47 [72] in the R environment v 4.1.3 [73]. The extracted bioclimatic variables were BIO1 (mean annual air temperature, °C), BIO2 (mean diurnal air temperature range, °C), BIO4 (temperature seasonality, °C/100), BIO5 (mean daily maximum air temperature of the warmest month, °C), BIO10 (mean daily mean air temperatures of the warmest quarter, °C), BIO12 (annual precipitation, kg m^−2^), BIO14 (precipitation in the driest month, kg m^−2^), BIO15 (precipitation seasonality, %), BIO17 (mean monthly precipitation in the driest quarter, kg m^−2^), BIO18 (mean monthly precipitation in the warmest quarter, kg m^−2^), and SWE (snow water equivalent, kg m^−2^) (Table S2). Elevation values were extracted from the REMA digital elevation model (100 m DEMs) [74] in QGIS Desktop 3.28.2 [75]. Distance from coast and ocean for each sample point (the latter relevant in the presence of floating ice shelves) was obtained using Bedmap2 raster files in QGIS (Table S2). Elevation and distance from coast/ocean data were used only for analyses focused on mainland samples. All maps reported in this work were created using QGIS Desktop 3.28.2 in Quantarctica [76].

### Sample classification into ACRBs

**Fig. 1:**
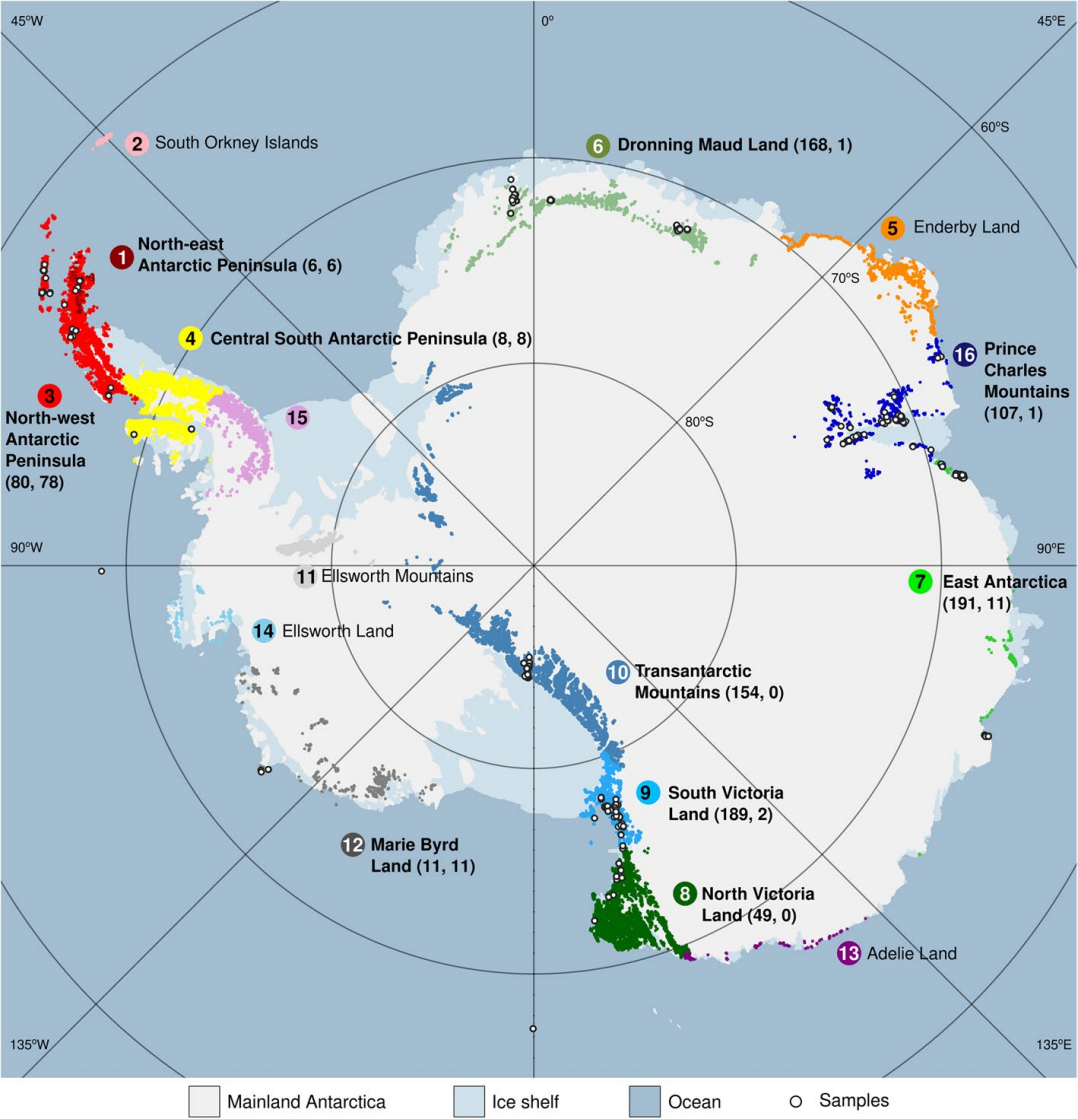
The Antarctic Conservation Biogeographic Regions (ACBRs) as defined in Terauds and Lee (2016). We examined a total of 988 samples from 10 ACBRs. These 10 ACBRs are shown in bold. ACBRs 2, 5, 11, 13, 14, and 15 were not represented in the sampling. Sample locations are indicated with white dots. The number of samples collected from each ACBR is reported in parentheses, followed by the number of samples collected from islands in each ACBR. Twenty-four samples collected from islands associated with mainland Antarctica, and sub- and peri-Antarctic islands are not included in the ACBR classification

Classically, terrestrial Antarctica is considered to include three broad biogeographic regions: the sub-, maritime, and continental Antarctic [9, 11]. Chown and Convey (2007) redefined the boundary between the latter two regions by their definition of the “Gressitt Line” as an important biogeographic boundary at the base of the Antarctic Peninsula [77]. More recently, the regions have been further divided into 16 distinct “Antarctic Conservation Biogeographic Regions” (ACBRs) [52, 53], a classification that applies specifically to the area of Antarctic Treaty governance south of the 60° latitude parallel. The samples in the 17 datasets represented here were obtained from 10 of the 16 currently recognized ACBRs: 1, 3, 4, 6–10, 12, and 16, as well as several of the sub- and peri-Antarctic islands (Fig. 1; Table S2) [58, 61, 78–85]. Our dataset includes 846 samples from the Antarctic mainland, 129 samples from islands and associated archipelagos of the Antarctic mainland, and 13 samples from the sub- and peri-Antarctic islands. Excluding samples from Bouvetøya, Peter I Øya, and Scott Island (accounting for 11 samples), all samples from the Antarctic mainland and its associated islands and archipelagos are included in the ACBR classification. The 118 samples obtained from islands and archipelagos associated with mainland Antarctica and included in the ACBR classification (ACBRs 1, 3, 4, 6, 7, 9, 12, and 16) are shown in Figure S1 and represent: James Ross Island (ACBR 1); South Shetland Islands (e.g., Livingston Island), Palmer Archipelago (e.g., Anvers Island), Adelaide Island and islands in its proximity, and Alectoria Island (ACBR 3); Alexander Island (ACBR 4); an un-named island in the Jelbart ice shelf (ACBR 6); Herring Island (Windmill Islands) and Hop Island (ACBR 7); Ross Island (ACBR 9); Siple, Lauff and Maher Islands (ACBR 12); and Samson Island (ACBR 16). The sub- and peri-Antarctic islands sampled included Marion, Possession, Kerguelen, Bartolomé, and South Georgia; sub- and peri-Antarctic islands are not included in the ACBR classification (Figure S2). Samples from these islands were considered together with those from Bouvetøya (formally included in the maritime Antarctic), Peter I Øya, and Scott Island (islands in the area of Treaty governance but not included in the ACBR classification) in the analyses described below and are referred to below as “ACBR unclassified islands” (AUI) for brevity. The AUI group does not aim to group islands on a bioregional basis; instead, it simply groups islands that did not fall into any ACBR to facilitate further analyses.

### Data analyses

All the 16S rRNA gene datasets except datasets 6 and 7 comprised raw forward and reverse reads. Data for datasets 6 and 7 (i.e., Victoria Land, Antarctic Peninsula and sub- and peri Antarctic island samples) were retrieved as pre-merged forward and reverse reads. For all other datasets, adapters were trimmed using Trimmomatic v 0.39 [86]. Each dataset was then separately analyzed in the R environment v 4.1.3 [73]. Sequences were processed with the same pipeline using dada2 v 1.22 [87]. Parameters “trimRight”, “trimLeft”, and “maxEE” from the function filterAndTrim() were set accordingly for the read length and error profile specific to each library (Table S3). Taxonomy was assigned using the SILVA database v 138.1 [88]. Blanks were present in datasets 1, 8, 10, and 16 and were removed using decontam v 1.14.0 [89]. All datasets were combined using the R package phyloseq v 1.38 [90]. Only sequences assigned to Bacteria, excluding mitochondrial and chloroplast DNA, were retained. Sequences assigned to the domain Archaea were excluded because previous tests demonstrated that the primer sets used in this study differentially targeted archaeal organisms [91]. Only samples with a sequence count of > 5000 were retained [92]. Due to the high disparity in sample read depth (*n* = 5247–988,658 reads) (Table S4), the dataset was normalized using the R package SRS v 0.2.3 [93] using the sample with the lowest amplicon counts (*n* = 5247) as the reference. Two samples in which all reads were assigned to unknown taxa were removed from the dataset after exploring their taxonomy as 100% of the reads were assigned to unknown taxa in those samples. Therefore, of the initial 1164 samples, 988 were used in further analyses. All of the following analyses were performed at the genus level because this composite dataset, comprising of several amplicon datasets spanning different 16S rRNA regions, did not allow accurate taxonomic analysis at the amplicon sequence variant (ASV) level [91]. Alpha diversity indices were generated using vegan v 2.6–4 [94] to calculate richness and Shannon indices on the genus-level taxonomic dataset. To test whether diversity indices differed across the defined regions, analysis of variance (ANOVA) was performed using the function aov followed, when significant, by Tukey’s honest significant difference (HSD) statistical tests to obtain pairwise comparisons. A conservative significance level of *p* < 0.01 was considered significant. ANOVA was only performed on bioclimatic variables from ACBRs 3, 6–10, and 16, and the ACBR unclassified islands (AUI) which were represented by at least 20 samples.

Permutational multivariate ANOVA (PERMANOVA) was performed using adonis2 (vegan), again using data only from ACBRs 3, 6–10, and 16, and the AUI which were represented by at least 20 samples. Pairwise comparison statistics were obtained using pairwiseAdonis v 0.4 [95], and *p* values were adjusted using the false discovery rate method (FDR) [96]. Both PERMANOVA and pairwise comparisons were calculated using 1000 permutations and calculated on Bray–Curtis dissimilarity matrices obtained from taxonomic datasets at the genus level, excluding reads that could not be classified at this level.

Bioclimatic variables were first standardized using the function decostand (vegan) and then checked for collinearity with vif.cca, and only variables with VIF < 20 were retained (maximum VIF retained = 12.33). The function ordiR2step was run to perform stepwise model building for distance-based redundancy analysis (dbRDA). The significance of the tested variables was obtained using anova.cca. Variation partitioning was performed with varpart on the same variables as selected from dbRDA. When variation partitioning was applied at the single ACBR level, only bioclimatic variables highlighted by dbRDA analysis performed on the entire dataset (BIO2, BIO4, BIO10, BIO15, and BIO18) were used. Geographical coordinates were transformed calculating principal coordinates of neighbor matrices using the function pcnm.

Principal Coordinate Analysis (PCoA) was performed using the pcoa function from the R library ape v 5.6–2 [97]. The function envfit (vegan) was then used to calculate ACBR and bioclimatic variable multiple regressions with the PCoA ordination axes. Only bioclimatic regions identified as significant by the function anova.cca were used in the envfit analysis.

Correlations between the genus-level taxonomic dataset and geographical distance (distance-decay) and bioclimatic dataset were assessed by calculating distance matrices using the vegan function vegdist for the community and bioclimatic datasets (Bray–Curtis and Euclidean distances, respectively) and distm for the geographical coordinates using the R library geosphere v 1.5–18 [98]. Mantel tests were then calculated using Spearman’s correlation and 1000 permutations. Dendrograms were created by calculating distance matrices as previously described and then by running the functions hclust and as.dendogram in the R package dendextend v 1.16.0 [99]. Dendrograms were combined with the function tanglegram.

Detailed taxonomic analyses were performed only on taxonomically consistent datasets derived from the amplification of the V3–V4 and V4 regions of the 16S rRNA gene (503 samples) [91]. This dataset subset represented 7 out of the 10 analyzed ACBRs (plus AUI) and contained samples from the Antarctic Peninsula, the Transantarctic Mountains, and the Victoria Land. No samples from Eastern Antarctica or Dronning Maud Land were included. The dominant community was defined as including all genera with a relative abundance of > 1% in at least one sample that was present in at least 10% of samples. The 10% threshold was chosen to include genera present exclusively on the Antarctic Peninsula. Random forest analysis was performed using the R package randomForest v 4.7 [100] on the genus-level dominant community dataset, which was transformed to relative abundance. Genera with “explained variance” higher than 30% using the randomForest algorithm were then used for Spearman’s semi-partial correlation with bioclimatic variables using the R packages ppcor v 1.1 [101] and rfPermute v 2.5.1 [102]. Correlations with *p* < 0.01 were considered significant. Only bioclimatic variables highlighted in dbRDA were used in these analyses to exclude collinear variables and therefore avoid redundancy (SWE, BIO2, BIO4, BIO10, BIO5, and BIO18). Genus relative abundances in each ACBR and the AUI were obtained by summing the reads of samples from the same ACBRs and then calculating relative abundances for each of them. Linear discriminant analysis effect size (LEfSe) analysis based on Kruskal–Wallis tests (*p* < 0.01) was performed on this data subset using the R package microeco v 1.1.0 [103]. Holm correction was used to adjust *p* values [104].

Figures were plotted using the R packages ggplot2 v 3.3.5 [105], gplots v 3.1.3 [106], gridExtra v 2.3 [107], and Cairo v 1.5.12.2 [108]. Data manipulation was carried out using default R packages and usedist v 0.4.0.9000 [109]. The R scripts used for the analysis of the sequencing data can be found on the GitHub page https://github.com/gvMicroarctic/AntarcticBiogeographyPaper.

## Results

### Alpha diversity and unclassified reads across ACBRs

A total of 52 bacterial phyla were identified from the Antarctic soils (Table S5). The most abundant phylum was Actinomycetota, accounting for 30.6% of reads, followed by Bacteroidota (13.9%), Pseudomonadota (13.5%), Chloroflexota (9.9%), Acidobacteriota (8.5%), Cyanobacteriota (5.1%), Verrucomicrobiota (3.4%), Gemmatimonadota (3.0%), Bacillota (2.7%), and Deinococcota (1.3%). One percent of reads were unclassified at the phylum level.

**Fig. 2:**
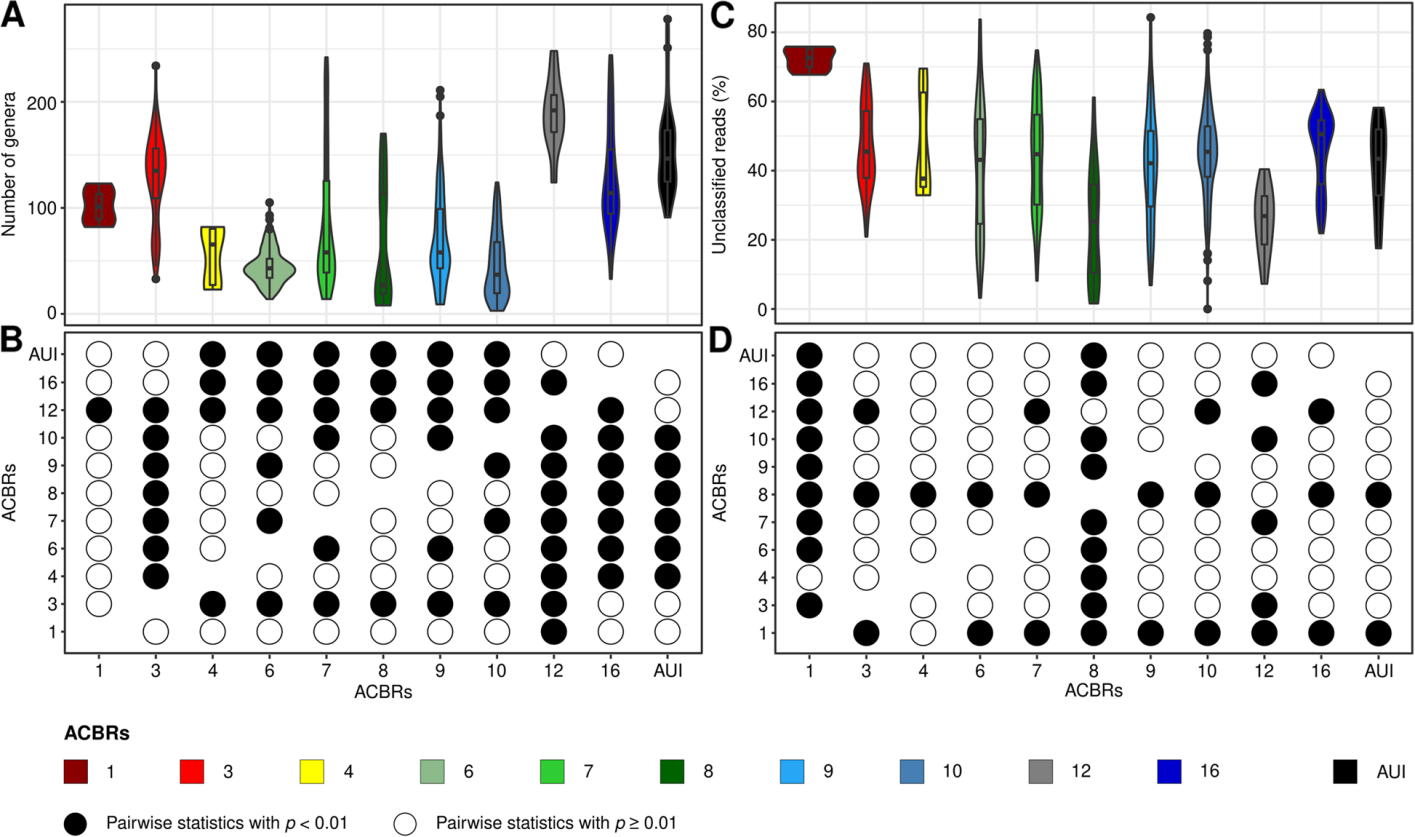
Number of bacterial genera and relative abundances of bacterial unclassified reads. Number of genera (i.e., richness) (**A**) and significant Tukey's statistical tests (*p* < 0.01) performed on the number of genera among ACBRs and AUI (**B**). Relative abundance of reads unclassified at the genus level (**C**) and significant Tukey's statistical tests (*p* < 0.01) performed on the relative abundance of unclassified reads among ACBRs and AUI (**D**). White circles correspond to non-significant Tukey’s statistical tests (*p* ≥ 0.01). ACBR 1: North-east Antarctic Peninsula; ACBR 3: North-west Antarctic Peninsula; ACBR 4: Central South Antarctic Peninsula; ACBR 6: Dronning Maud Land; ACBR 7: East Antarctica; ACBR 8: North Victoria Land; ACBR 9: South Victoria Land; ACBR 10: Transantarctic Mountains; ACBR 12: Marie Byrd Land; ACBR 16: Prince Charles Mountains. AUI: ACBR unclassified islands

The total number of genera included in the dataset was 1445, ranging from 3 to 278 across individual samples (Table S6). The highest numbers of genera were recorded from the AUI and in ACBRs 1, 3, 12, and 16, while ACBRs 4 and 6–10 had the lowest numbers of genera (Fig. 2A). The numbers of genera differed significantly between ACBRs (ANOVA *F* = 56.84, df = 10, *p* < 2e − 16). High numbers of Tukey’s pairwise comparisons (from 7 to 9) were significant (*p* < 0.01) for ACBRs 3, 12, and 16, while ACBRs 1, 4, and 8 showed the lowest numbers of significant pairwise differences (from 1 to 4) (Fig. 2B). Similar trends were observed for Shannon diversity (ANOVA *F* = 66.71, df = 10, *p* < 2e − 16), although fewer significant pairwise correlations were obtained, suggesting less divergent Shannon diversity across ACBRs (Figure S3). Because alpha diversity indices were calculated at the genus level, low richness and Shannon diversity values for some ACBRs could result from samples with high “unknown” counts (Table S6). The percentage of reads assigned to unknown genera varied across samples, ranging from 0.0 to 84.3% (Fig. 2C). This potential bias was confirmed by a significant Pearson’s correlation between the total number of genera and the percentage of unknown genera (*r* =  − 0.1624, *p* = 2.858e − 07).

Alpha diversity showed significant correlations with bioclimatic variables, with significant positive correlations for BIO1, BIO5, BIO10, BIO12, BIO14, BIO17, BIO18, and SWE, and negative correlations for BIO2, BIO4, BIO15, and elevation, for both Shannon diversity and richness (Figures S4–S5). Distance to the ocean was significantly correlated only with Shannon diversity (Figures S4–S5).

### ACBR clustering

**Fig. 3:**
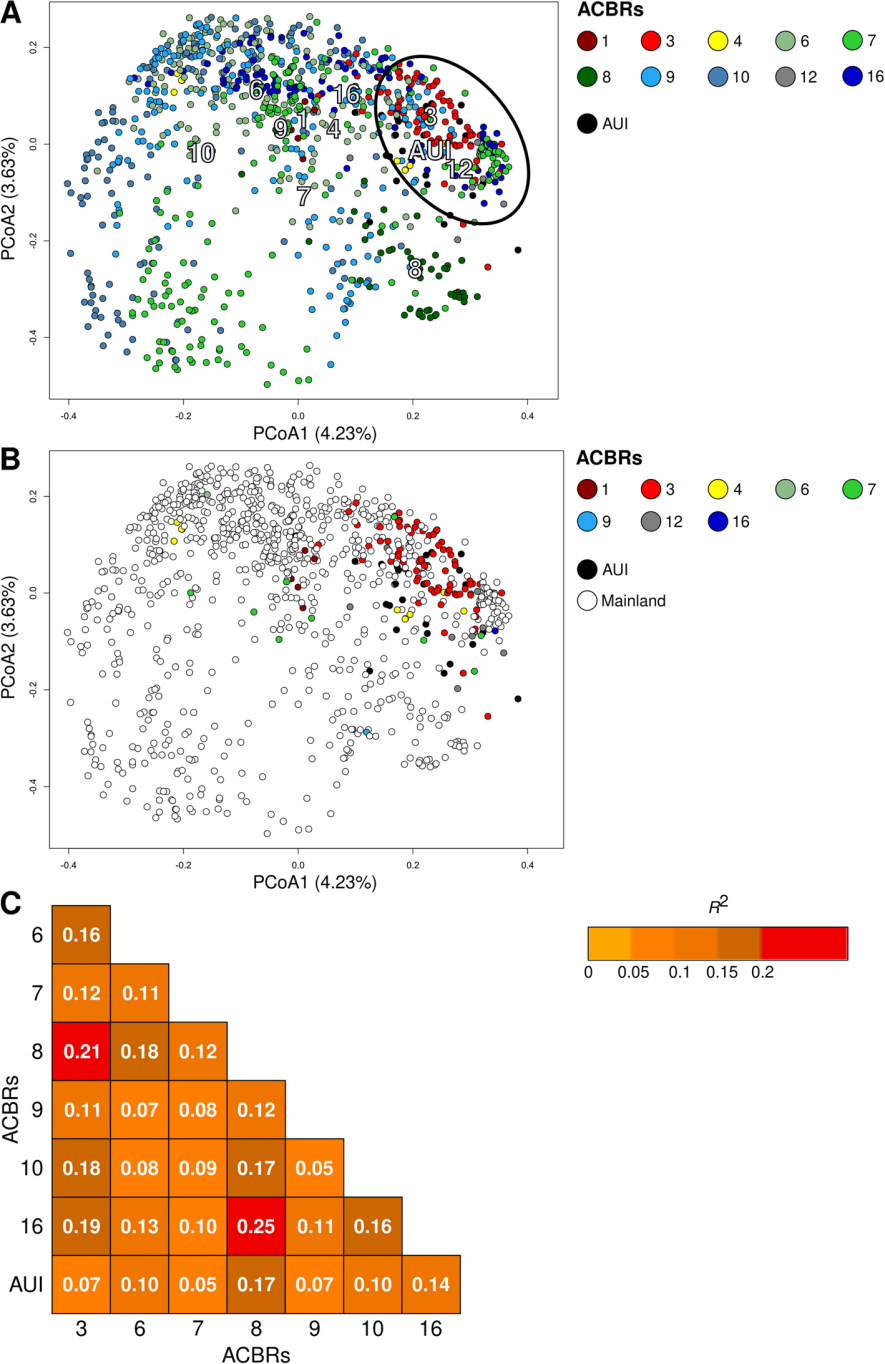
Antarctic soil bacterial community beta-diversity. Principal Coordinate Analysis (PCoA) was calculated on the Hellinger-transformed bacterial community at the genus level where all samples (**A**) and only island samples (**B**) are colored as the respective ACBRs. ACBRs were plotted in the PCoA plot following envfit test (**A**). PERMANOVA pairwise comparisons (**C**) were performed only between ACBRs represented by at least 20 samples. PERMANOVA was performed on Bray–Curtis dissimilarity matrices calculated on the Hellinger-transformed bacterial community at the genus level (1000 permutations). All the reported pairwise comparisons are statistically significant (adjusted *p* < 0.01). ACBR 1: North-east Antarctic Peninsula; ACBR 3: North-west Antarctic Peninsula; ACBR 4: Central South Antarctic Peninsula; ACBR 6: Dronning Maud Land; ACBR 7: East Antarctica; ACBR 8: North Victoria Land; ACBR 9: South Victoria Land; ACBR 10: Transantarctic Mountains; ACBR 12: Marie Byrd Land; ACBR 16: Prince Charles Mountains. AUI: ACBR unclassified islands

Soil bacterial communities from AUI and ACBRs 3 and 12 clustered together in the PCoA plot (Fig. 3A). Samples from ACBR 8 showed a distinct clustering, suggesting consistent bacterial community structures within that ACBR (Fig. 3A). Samples from all other ACBRs did not show clear clustering according to region. ACBRs 1, 3, and 4 differed geographically from other mainland ACBRs, as their samples were mainly represented by soils collected from islands close to the Antarctic Peninsula. In terms of soil bacterial community structure, ACBR 3 samples were more closely related to those from sub- and peri-Antarctic islands (i.e., part of AUI; Fig. 3B).

Despite the high level of overlap between soil bacterial communities, different ACBRs could be significantly distinguished using a Bray–Curtis dissimilarity matrix (PERMANOVA *R*^2^ = 0.19, *p* < 0.01). Pairwise comparisons showed that soil bacterial communities from ACBRs 3 and 8 were the most distinct (Fig. 3C). AUI showed the lowest degree of dissimilarity to ACBRs 3, 7, and 9, all of which themselves included soil samples collected from islands.

**Fig. 4:**
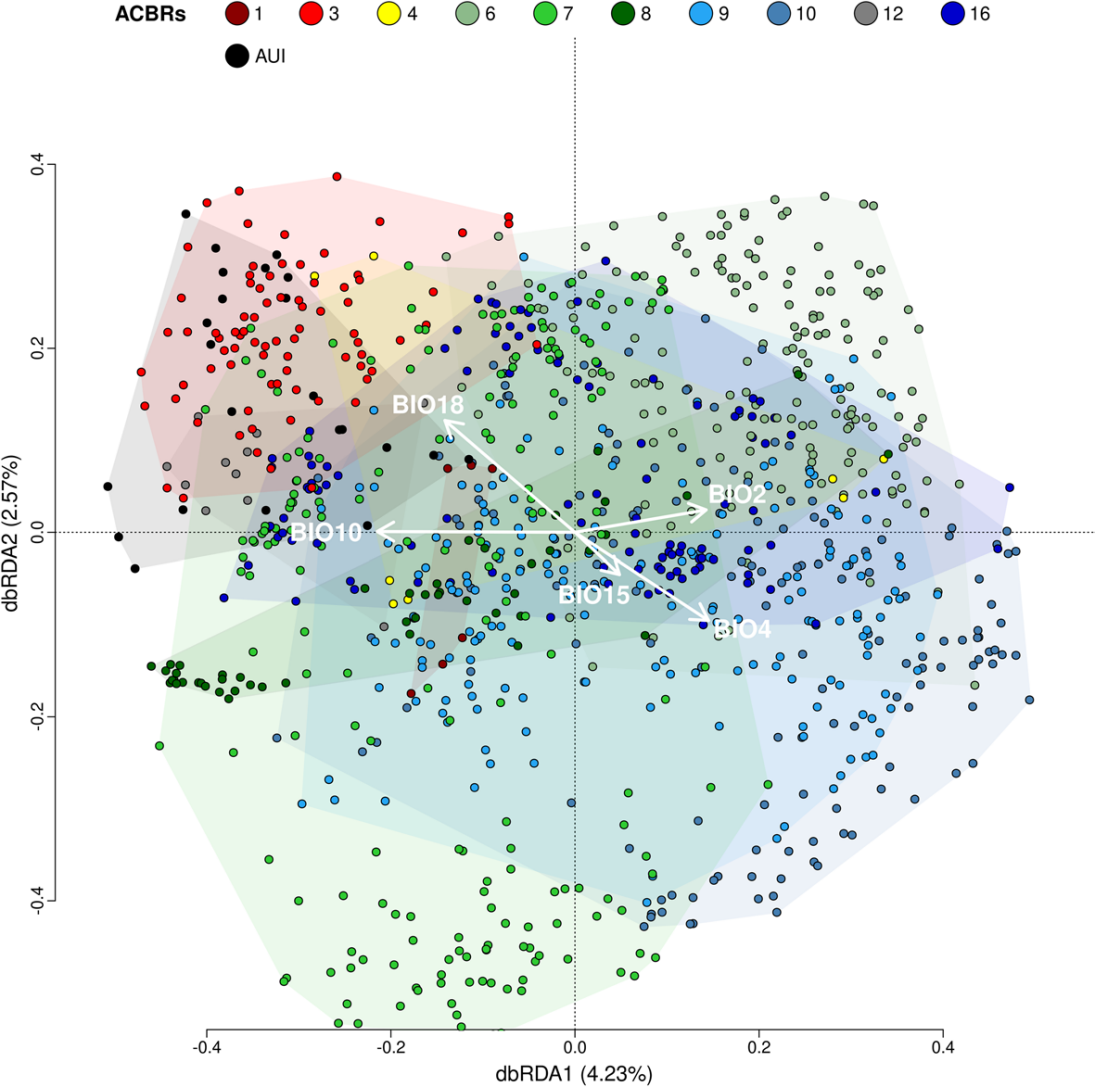
Distance-based redundancy analysis (dbRDA) performed on the Hellinger-transformed genus and standardized bioclimatic variable datasets. BIO2: mean diurnal air temperature range; BIO4: temperature seasonality; BIO10: mean daily mean air temperatures of the warmest quarter; BIO15: precipitation seasonality; BIO18: mean monthly precipitation amount of the warmest quarter; ACBR 1: North-east Antarctic Peninsula; ACBR 3: North-west Antarctic Peninsula; ACBR 4: Central South Antarctic Peninsula; ACBR 6: Dronning Maud Land; ACBR 7: East Antarctica; ACBR 8: North Victoria Land; ACBR 9: South Victoria Land; ACBR 10: Transantarctic Mountains; ACBR 12: Marie Byrd Land; ACBR 16: Prince Charles Mountains. AUI: ACBR unclassified islands

dbRDA only explained 12.0% (adjusted *R*^2^) of the observed variance in community structure across the dataset (Fig. 4). Bioclimatic variables related to temperature (BIO10, mean daily mean air temperatures of the warmest quarter) and precipitation (BIO18, mean monthly precipitation amount of the warmest quarter) were drivers for the bacterial community composition in ACBRs 1, 3, 12, and AUI; most of these samples were obtained from Antarctic, sub-Antarctic, and peri-Antarctic islands. Bioclimatic variables related to precipitation and temperature seasonality and daily ranges (BIO2, BIO4, and BIO5) were drivers for the bacterial communities in ACBRs 9 and 10; most samples from these ACBRs were sampled from mainland Antarctica. Sample distributions partially followed bioclimatic conditions in the different ACBRs. ACBRs 1, 3, 4, 7, and 12 have higher mean daily air temperatures in the warmest quarter (BIO10) and mean monthly precipitation amounts in the warmest quarter (BIO18) compared to ACBRs 6, 8–10, and 16 (Figure S6; Table S2). Mean diurnal air temperature range, temperature, and precipitation seasonality (BIO2, BIO4, and BIO15, respectively) were lowest in ACBRs 1, 3, and 12 compared to the other ACBRs (Figure S6). All bioclimatic variables showed statistically significant differences (*p* < 0.01) between ACBRs; this was also the case for elevation, “distance from the ocean” and “distance from the coast” (Table S7). ACBR 4 showed mixed characteristics with high temperatures but also high ranges and seasonality values (Figure S6); this was reflected in the dbRDA, in which ACBR 4 samples showed a wide clustering in the plot (Fig. 4).

Distance-decay analyses showed that geographic distance between samples exhibited a low, but significant, correlation (*r* = 0.1499, *p* = 0.0009) with bacterial composition dissimilarity scores between samples (Figure S7A). Variation partitioning also showed that 13.2% of bacterial community variation was explained by geographical distance between samples alone, while only 2.7% of the variation was explained by the bioclimatic data, with 6.6% of variance being explained by their interaction (Figure S8A) at a significance threshold of *p* < 0.01 (Table S8).

### Bacterial community structure explained by ACBRs

**Fig. 5:**
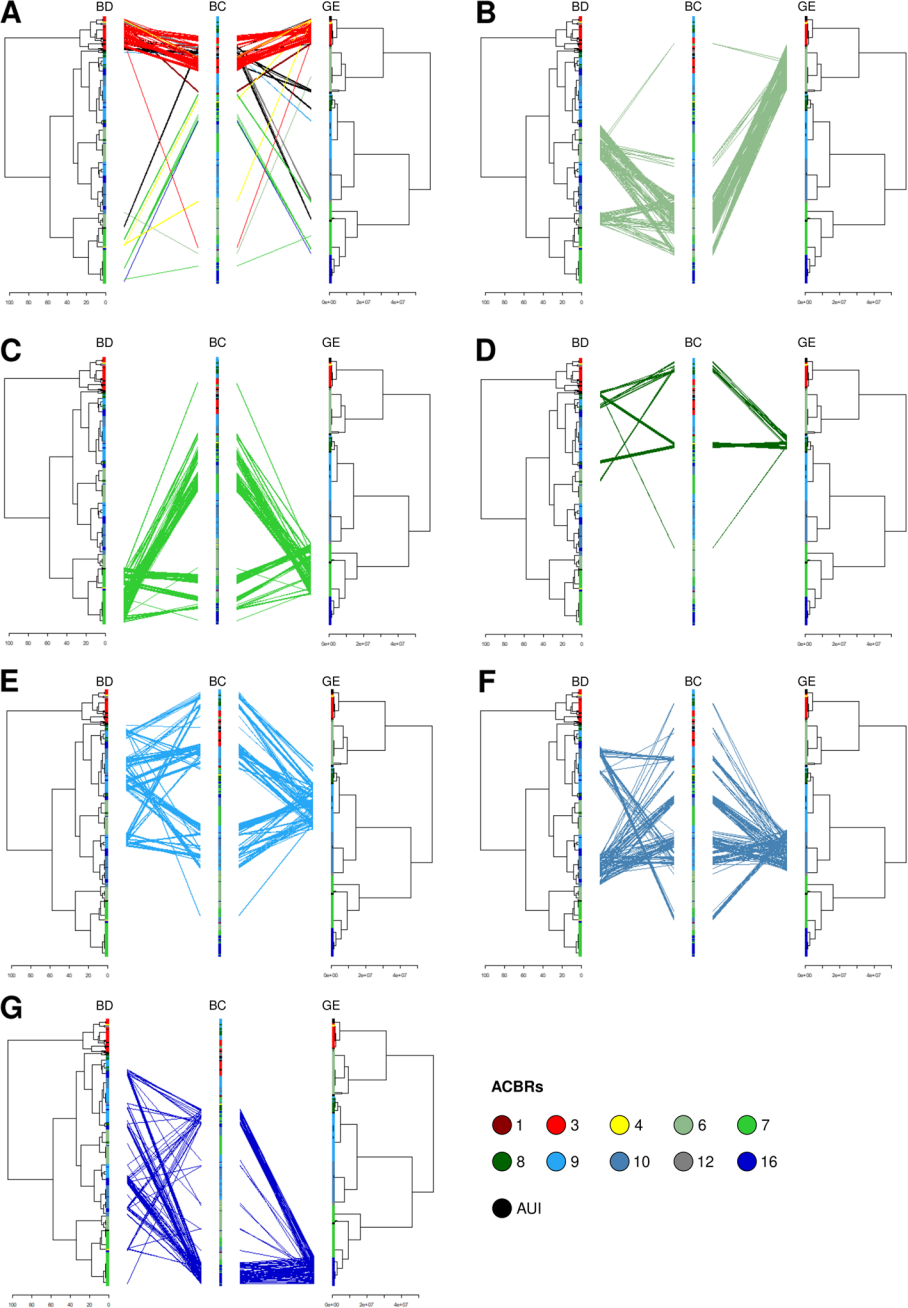
Sample clustering at bioclimatic, bacterial community, and geographic level. Tanglegrams representing bioclimatic data (BD), bacterial community (BC), and geography (GE) for samples collected from islands (**A**), ACBR 6 (**B**), ACBR 7 (**C**), ACBR 8 (**D**), ACBR 9 (**E**), ACBR 10 (**F**), and ACBR 16 (**G**). Geography: geographical distances between samples in the form of latitude and longitude information; Bacterial community: Hellinger-transformed community at genus level; Bioclimatic data: BIO1, BIO2, BIO4, BIO5, BIO10, BIO12, BIO14, BIO15, BIO17, BIO18, and SWE associated to each sample. ACBR 1: North-east Antarctic Peninsula; ACBR 3: North-west Antarctic Peninsula; ACBR 4: Central South Antarctic Peninsula; ACBR 6: Dronning Maud Land; ACBR 7: East Antarctica; ACBR 8: North Victoria Land; ACBR 9: South Victoria Land; ACBR 10: Transantarctic Mountains; ACBR 12: Marie Byrd Land; ACBR 16: Prince Charles Mountains. AUI: ACBR unclassified islands

The most consistent clustering of samples was detected for ACBR 3 (Fig. 5A and S9), where samples largely clustered together for both abiotic variables and soil bacterial community structures (Fig. 5A). Whereas samples collected from islands showed similar bacterial communities for ACBRs 3, 12, and AUI, other island samples clustered more closely to soils taken from the same ACBR compared to samples collected from other islands, as in the cases of ACBR 7 and 16 (Fig. 5A). Samples from other ACBRs followed different trends (Fig. 5B–G). For example, we observed a consistent clustering of samples from ACBR 6 according to bacterial composition and geographical trends, whereas the bioclimatic data did not show a detectable clustering (Fig. 5B). By comparison, relatively consistent geography was shown for samples from ACBRs 8–10 and 16, but samples were characterized by variable bioclimatic data, which was reflected by a wider bacterial community distribution (Fig. 5D–G). Samples from ACBR 7 were consistent both in geography and bioclimatic data but the bacterial community formed separate clusters (Fig. 5C). These clusters partially corresponded to samples from the Windmill Islands and Vestfold Hills regions (Figure S10) and PERMANOVA performed using this grouping generated a significant outcome (*p* < 0.01) with *R*^2^ = 0.14. Samples from the Vestfold Hills clustered more closely to those collected from ACBRs 9 and 10, whereas samples from the Windmill Islands clustered more closely to those from ACBR 3 and AUI.

AUI and ACBRs 8 and 10 showed higher variance explained by bioclimatic data, while ACBRs 3, 6, 7, 9 and 11 showed higher variance explained by geography (Figure S8B–I). Variation partitioning performed on AUI and ACBR 8 showed a lower residual variance not explained by bioclimatic variables and geography compared to the other ACBRs (0.42 and 0.362, respectively). The ACBRs with the highest residual component of variance were ACBRs 9 and 16 (0.816 and 0.879, respectively). The other ACBRs showed residual variance between 0.662 and 0.764.

### Islands vs mainland

PERMANOVA performed on the dissimilarity matrices of soil bacterial community structures of island samples vs mainland samples was significant, although significance scores were lower than for ACBRs (*p* = 0.0009, *R*^2^ = 0.0361). This indicated that only a low percentage of observed variance was explained by whether a sample was collected from the Antarctic mainland or associated islands and is consistent with our previous observations that island soil microbiomes were more similar to geographically less distant soils than to soils collected from other islands (Fig. 3B and 5A).

**Fig. 6:**
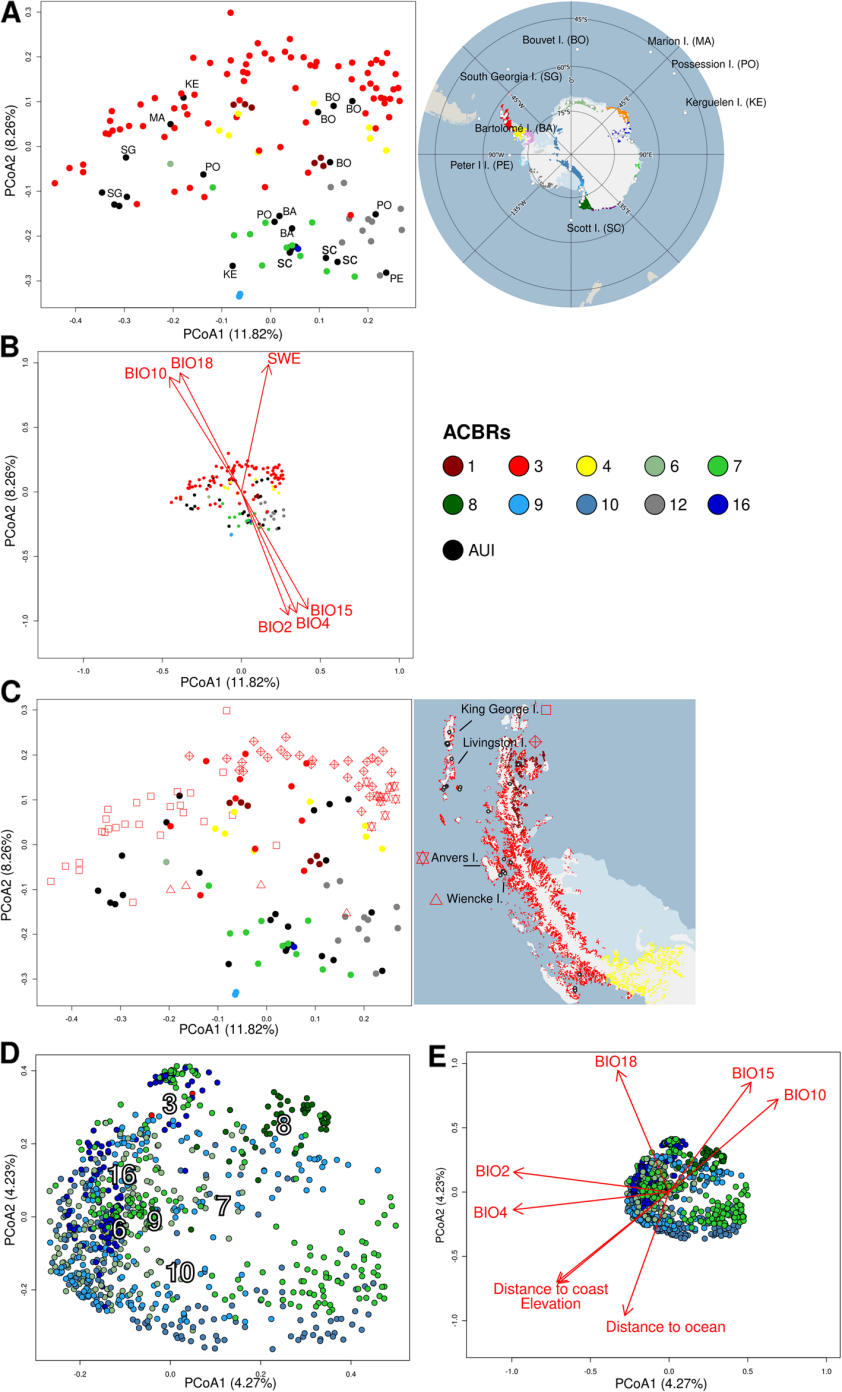
Principal Coordinate Analysis (PCoA) calculated using only island or mainland samples. PCoA calculated on the Hellinger-transformed bacterial community at genus level from samples collected from islands. PCoA highlighting samples collected from islands not classified in the ACBR classification of Terauds et al. (2012) (**A**), reporting community correlation with bioclimatic variables (**B**), and highlighting samples from ACBR 3 and with more than two samples per island (**C**). PCoA calculated on the Hellinger-transformed bacterial community at genus level from samples collected from the mainland showing ACBRs (**D**) and bioclimatic variables (**E**) correlations to the bacterial dataset were calculated using envfit. BIO2: mean diurnal air temperature range; BIO4: temperature seasonality; BIO10: mean daily mean air temperatures of the warmest quarter; BIO15: precipitation seasonality; BIO18: mean monthly precipitation amount of the warmest quarter; SWE: Snow water equivalent; ACBR 1: North-east Antarctic Peninsula; ACBR 3: North-west Antarctic Peninsula; ACBR 4: Central South Antarctic Peninsula; ACBR 6: Dronning Maud Land; ACBR 7: East Antarctica; ACBR 8: North Victoria Land; ACBR 9: South Victoria Land; ACBR 10: Transantarctic Mountains; ACBR 12: Marie Byrd Land; ACBR 16: Prince Charles Mountains. AUI: ACBR unclassified islands

We also identified a division between islands from ACBRs 1, 3, and 4 (islands from the Antarctic Peninsula) and all the other islands (Fig. 6A). Islands that were not grouped into the ACBR classification (i.e., the AUI) also showed distinct clustering: soil bacterial communities from the islands of South Georgia, Bouvet, and Marion islands all clustered with those from Antarctic Peninsula islands, whereas Peter Øya, Scott, and Bartolomé islands grouped closer to islands from ACBRs 7, 9, 12, and 16 (except for two samples collected from Possession and Kerguelen islands, which clustered close to Antarctic Peninsula samples) (Fig. 6A). Samples from the Antarctic Peninsula islands (ACBRs 1, 3, and 4) correlated significantly (*p* < 0.01) with all temperature- and precipitation-related bioclimatic variables (BIO10, BIO18, and SWE), while the other islands correlated with seasonal or diurnal differences in precipitation or temperature (BIO2, BIO4, and BIO15) (Fig. 6B and S11A). It is appropriate to note that ACBR 3 contained the highest number of island samples in our dataset, and these showed a strong clustering of bacterial composition according to the island from which the samples were collected (Fig. 6C). This consistent clustering by island was confirmed by PERMANOVA with just the island dataset (*p* = 0.0009, *R*^2^ = 0.53) when using the island as the explanatory factor for differences in bacterial composition between samples. By comparison, the *R*^2^ value was 0.04 when ACBR was used as an explanatory factor for the island dataset.

Grouping mainland samples by factor “ACBR” also resulted in a weak (*R*^2^ = 0.025), albeit statistically significant correlation (*p* < 0.01). Stronger correlations were identified when Mantel tests were used to compare bacterial community dissimilarities and geography (distance-decay, with* r* = 0.1611, *p* = 0.0009), and bacterial community dissimilarities and environmental variables (*r* = 0.1102, *p* = 0.0009) (Figure S7E–F). By comparison, Mantel tests for differences between bacterial community dissimilarities and geography for the island dataset yielded *r* = 0.37776 (*p* = 0.0009), and *r* = 0.3001 (*p* = 0.0009) for bacterial community dissimilarities and environmental variables (Figure S7C–D). When Mantel tests were used to compare mainland bacterial community and elevation, distance from the coast, or distance from the ocean, only elevation correlated significantly (*r* = 0.0780, *p* = 0.0009) (Figure S7G–I). The separate PCoA clustering observed for ACBR 8 might be due to negative correlations with elevation and distances from the coast/ocean of ACBR 8 samples (Fig. 6D–E and S8B).

When only the island dataset was analyzed, 19.1% of the variance was explained by geography, 9.2% by bioclimatic data, 4.6% by their interaction, and 67.1% was residual (Figure S8J). When only mainland samples were analyzed, variation partitioning analyses showed 78.9% residual variance, with geographic distances explaining the most variance in bacterial community structure between samples (geography = 9.8%, bioclimatic data = 4.0%, elevation/distance from coast/ocean = 1.3%) (Figure S8K).

### Climatic drivers of the dominant soil bacteria and indicator taxa across Antarctica

**Fig. 7:**
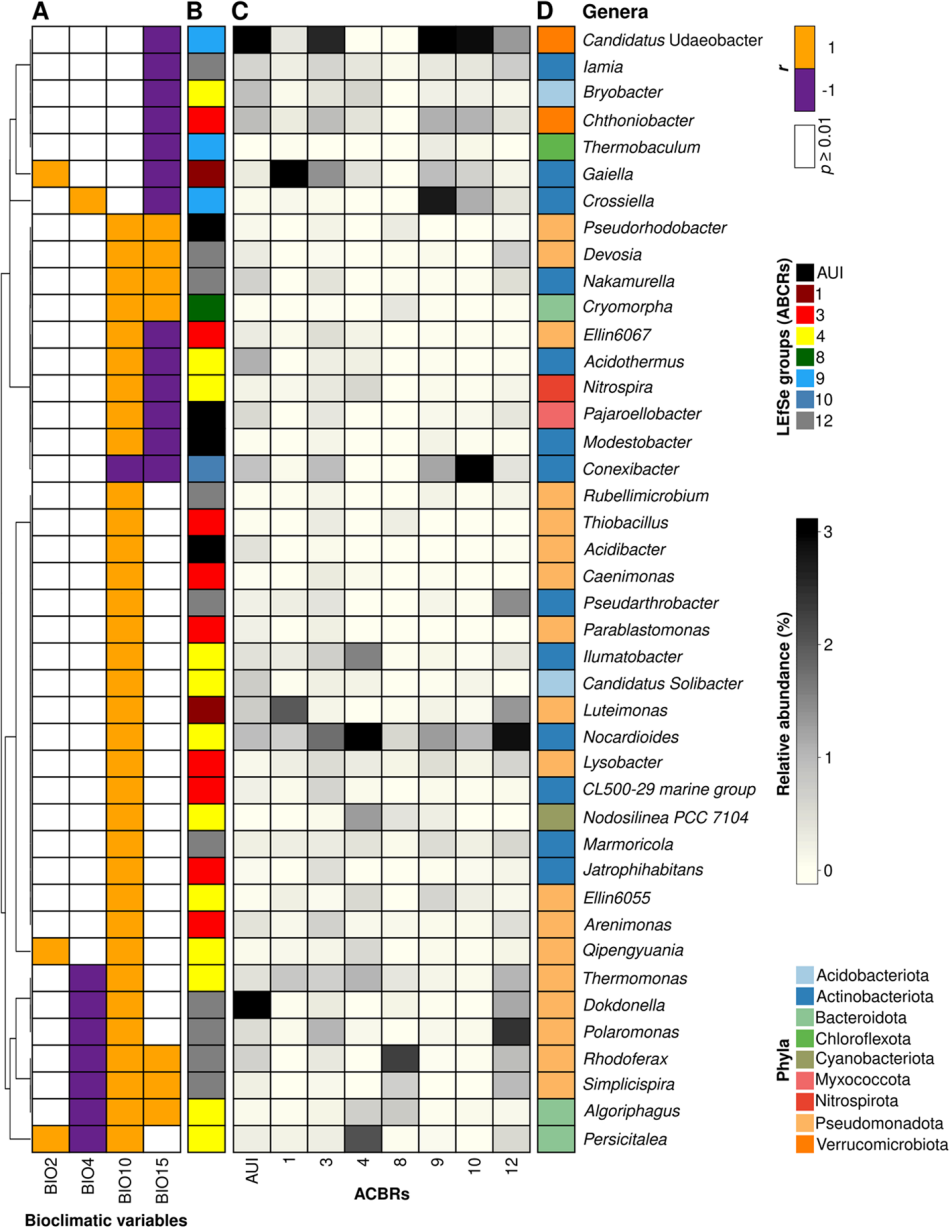
Random forest analysis and abundance of selected genera. Semi-partial Spearman’s correlations between relative abundances of dominant genera selected by the random forest analysis (variance explained > 30%) and bioclimatic variables (**A**). Indicator taxa across ACBRs and AUI (LEfSe analysis based on significant (*p* < 0.01) Kruskal–Wallis tests) (**B**). The relative abundance of dominant genera selected by the random forest analysis, with the reported relative abundances are represented by ACBR (**C**), with associated phylum taxonomic assignment (**D**). Dominant genera were defined as those with a relative abundance of > 1% in at least one sample that were present in at least 10% of samples. Only samples sequenced with V3*–*V4 and V4 16S rRNA primers were used for this analysis to ensure the best taxonomic consistency between samples (Varliero et al., 2023). Correspondingly, this approach included samples from AUI (“ACBR unclassified islands “) and ACBRs 1, 3, 4, 8, 9, 10, and 12. BIO2: mean diurnal air temperature range; BIO4: temperature seasonality; BIO10: mean daily mean air temperatures of the warmest quarter; BIO15: precipitation seasonality; BIO18: mean monthly precipitation amount of the warmest quarter; SWE: Snow water equivalent

In order to understand how climatic variables impacted the more abundant bacterial taxa in Antarctic soil communities, we performed a correlation analysis between the bioclimatic variables and the relative abundance of the dominant genera across the sample sets. A total of 149 genera were identified as dominant (i.e., present in more than 10% of samples and with a relative abundance higher than 1% in more than one sample). Of these, 51 genera showed habitat preferences linked to the tested bioclimatic variables based on random forest predictions (Figure S12). Semi-partial correlations performed on the 51 genera and the six bioclimatic variables showed that most of the significant correlations were linked to mean daily mean air temperatures of the warmest quarter (BIO10, 35 correlations), followed by precipitation seasonality (BIO15, 20 correlations), temperature seasonality (BIO4, eight correlations) and mean diurnal air temperature range (BIO2, three correlations). No significant correlations (*p* ≥ 0.01) were found for snow water equivalent (SWE) and mean monthly precipitation amount of the warmest quarter (BIO18) (Fig. 7A). All of the dominant genera presented in Fig. 7 showed habitat preferences correlated with BIO10, except for *Crossiella, Gaiella*, *Thermobacum*, *Chthoniobacter*, *Bryobacter*, *Iamia*, and *Candidatus* Udaeobacter, which showed negative correlations with BIO15. All correlations between genera and BIO10 were positive, except for *Conexibacter* which showed a negative correlation with BIO10. Thirteen of the genera that significantly correlated with BIO10 also correlated with BIO15. Of the eight genera correlating with BIO4, seven showed a negative correlation (*Persicitalea, Algoriphagus, Simplicispira, Rhodoferax, Polaromonas, Dokdonella,* and *Thermomonas*) and one showed a positive correlation (*Crossiella*). Three genera showed positive correlations with BIO2 (*Persicitalea, Qipengyuania, and Gaiella*) (Fig. 7A). All of the genera identified in the random forest analysis with at least one positive or negative correlation to the tested bioclimatic variables were also identified as indicator taxa for the different ACBRs using LEfSe analysis. The highest numbers of indicator taxa which also correlated to bioclimatic variables were in ACBR 4 (12 indicators), ACBR 12 (10), ACBR 3 (9), AUI (4), and then ACBRs 1, 8, 9, and 10 reporting only between 1 and 3 indicator taxa (Fig. 7B). The highest number of bacterial indicators was therefore ascribed to maritime Antarctica, corresponding to the areas where the highest diversity of genera was observed (Fig. 2). The most abundant genera that showed correlations to climatic parameters and were also identified as indicator taxa were *Conexibacter* (0.1–8.7%), *Gaiella* (0.0–4.2%), *Nocardioides* (0.5–3.8%), *Candidatus* Udaeobacter (0.0–3.2%), and *Dokdonella* (0.0–3.2%) (Fig. 7C; Table S9). The most abundant genera that were not selected by random forest modeling (variance explained < 30%) were *Nostoc* (0.0–7.0% in the different ACBRs), *Tychonema* (0.0–6.3%), *Blastocatella* (0.8–5.6%), *Pedobacter* (0.6–5.1%), and *Sphingomonas* (0.8–3.4%) (Figure S13).

## Discussion

Our data reveal that bacterial communities in Antarctic soils do not align closely with the ACBR characterization proposed by Terauds et al. and Terauds and Lee [52, 53]. Only 19% of the variability was explained by ACBR as a discriminating factor, with high unexplained residual variability. This percentage further decreased (to 2.5%) when Antarctic-associated island soil samples were removed from the dataset (although this also had the confounding effect of removing most samples obtained from maritime Antarctica), supporting the conclusion that Antarctic mainland soil microbial community structures poorly reflect the ACBR classification, which itself is largely based on diversity patterns of macroscopic organisms. The most significant clustering in our dataset was between samples collected from either the continental Antarctica or the maritime Antarctica (the Antarctic Peninsula and the large off-shore archipelagoes of the South Shetland Islands and South Orkney Islands). Within these two areas, microbial communities showed high structural homogeneity, therefore not closely reflecting the ACBR classification [52, 53]. Bacterial communities from North Victoria Land (ACBR8) represented an exception, as they showed the highest microbial compositional differences compared to all the other ACBRs in continental Antarctica, possibly due to the fact that ACBR 8 experiences higher temperatures and precipitation rates compared to the remainder of the Victoria Land region (Figure S6). The observed clustering was principally explained by bioclimatic conditions, where samples from AUI and ACBRs 1, 3, 4, and 12 showed higher precipitation and temperature values (BIO10 and BIO18), and lower daily and seasonal precipitation and temperature ranges (BIO2, BIO4 and BIO15). It is probable that soil bacterial communities in these ACBRs are subject to more favorable environmental conditions than those in ACBRs 6–10 and 16 [110]. More challenging environmental conditions impose increased selection for resistant and/or resilient microbial communities [111]. These observations are consistent with the observed reductions in Antarctic soil bacterial diversity at higher latitudes [112], which are largely explained by reductions in air temperature and water availability [57, 113]. This was also supported by the observed correlations between air temperature, and other bioclimatic variables, with bacterial diversity: soils subjected to higher air temperatures and precipitation showed higher bacterial diversity, whereas those sampled from environments with wider seasonal and daily changes in temperature and precipitation showed lower diversity (Figure S4–S5). This suggests that bacterial diversity is higher under more favorable environmental conditions (higher water availability, higher temperatures, and more stable environmental conditions) and lower in more life-challenging conditions [57]. As temperature and precipitation regimes influence water and nutrient bioavailability [114], microbiome functionality [115, 116], and diversity [57] in soil, they are logical explanatory factors (SWE, BIO2, BIO4, BIO10, BIO15, and BIO18) of observed Antarctic soil bacterial community patterns (Figs. 3 and 4 and Figure S11). Nevertheless, only a low percentage of variability overall was explained by bioclimatic variables (Figs. 3 and 4, Figures S8 and S11).

A high percentage of unexplained variance is observed both when ACBR classification and bioclimatic variables are taken into consideration as explanatory factors. This is not surprising as microbial communities on the Antarctic continent are distributed and structured by a multiplicity of environmental factors and dynamics. Distance-decay relationships between geographical distances and microbial diversity vary in relation to environmental connectivity [17], where the Antarctic continent includes both connected and fragmented habitats [17, 81], and therefore presents a diversity of factors that shape microbial distributions in different Antarctic areas. Notably, it is currently thought that eolian microbial dispersal in continental Antarctica is limited [56, 117] and that microbial community distribution patterns in Antarctica are more influenced by the existence of suitable glacial refugia (habitable areas that persisted during glaciation cycles) and therefore by soil histories and climate legacies [37, 118]. A regional study conducted in the McMurdo Dry Valleys showed that soil microbial composition was impacted by geographical distance, in part due to variations of the geochemical variables across the studied region [39]. In the Transantarctic Mountains, microbial diversity in soil ecosystems significantly varies with terrain age, illustrating that biotic communities may vary with both abiotic spatial heterogeneity and geological history [119]. At continental scales, geographical distances can shape microbial distributions due to dispersal patterns, climatic characteristics, and continental formation history [7, 14]. The lack of available consistent geochemical data for our datasets probably also contributed to the observed high percentage of unexplained variance. It has frequently been observed that Antarctic soil microbial distribution varies in relation to edaphic characteristics such as pH, electrical conductivity, soil moisture, soil temperature, and nutrient content [20, 30, 57, 58, 120, 121]. However, climate influences edaphic characteristics, therefore indirectly influencing soil microbial communities [122].

Our results suggest that the geographically large ACBRs may be too broad to capture microbial biodiversity patterns as they encompass regions that have very different environmental conditions and, thus, soil bacterial communities. A pertinent example in the current study is that of ACBR 7, in which distinct clustering separating the Windmill Islands and the Vestfold Hills was observed. These regions represent very different environments [7], with the Vestfold Hills being more extreme and dominated by Actinobacteriota, and the near-coast Windmill Islands having unique microbial communities with some sites dominated by *Candidatus* Dormibacteraeota and Eremiobacteriota [7]. Notably, the bacterial communities of the Vestfold Hills clustered more closely with samples collected from ACBRs 9 and 10, also characterized by extreme climatic conditions, compared to ACBR 3 and the sub- and peri-Antarctic islands which clustered with samples collected from the Windmill Islands region (Figures S6 and S10).

We also identified a strong divergence in the drivers of soil bacterial community composition when considering specifically the Antarctic/sub-Antarctic islands and the continental mainland. For instance, bacterial communities clustered consistently within each island/island group examined, but did not show any clear geographical differentiation on the Antarctic mainland. This might be because the island and mainland soils have different formational histories [5]. Island bacterial communities were generally distinct from each other, highlighting the strong effect of island isolation on bacterial community development [123]. A previous study reported changes between inland and coastal soils in terms of fungal diversity due to differences in soil geochemistry and environmental conditions [124]. Because microbial community distributional patterns are partially shaped by bioclimatic conditions, and because observations and model predictions highlight that maritime Antarctica is most strongly affected by global warming [125–127], microbial community distribution and diversity in this region may be highly impacted in the near future [128].

Some of the sampled areas in our dataset are not included in the ACBR classification. These included remote oceanic islands included within the maritime Antarctic (Bouvetøya, Peter I Øya) and the continental Antarctic near-coast (Scott Island), and the sub- and peri-Antarctic islands (South Georgia and Marion, Possession, Kerguelen and Bartolomé islands). Soil bacterial communities from these islands clustered more closely with geographically associated islands/groups within the ACBR system, and soils from South Georgia Island, Marion Island, and Bouvetøya clustered closely with those from the maritime Antarctic ACBRs 1, 3, and 4 (Group 1), whereas soils from Scott Island, Peter I Øya, and Bartolomé Island clustered closer to the continental Antarctic islands (from ACBRs 7, 9, and 12) (Group 2). Group 2 might indicate the influence of the Pacific Ocean and currents around the entire continental Antarctic coastline. Furthermore, the Antarctic Coastal current flows south along the western Antarctic Peninsula (Group 1), potentially influencing soil microbiomes on the South Shetlands and maritime Antarctic coastline (also Group 1). A similar argument was previously advanced by Pugh and Convey (2008) [28], but more studies are needed to test this hypothesis. Lebre et al. (2023) have proposed a differentiation of the sub- and peri-Antarctic islands presented in this study into the “classical” maritime, continental, and sub-Antarctic regions, based on soil microbial community compositions [59]. However, the addition of maritime Antarctic Islands from the Antarctic Peninsula region in the current study is not consistent with this proposed island clustering (c.f., [59]), further highlighting the importance of spatial scales when studying patterns of soil microbial ecology [58].

Of the dominant genera, 28% showed habitat preferences connected to at least one of the bioclimatic variables, particularly for habitats with higher temperatures (BIO10) and precipitation seasonality (BIO15) (Fig. 7A). Some of the genera showing habitat preferences, such as *Nitrospira* and *Thiobacillus*, have important ecosystem functions. Members of *Nitrospira* and *Thiobacillus* are chemolithotrophic nitrite- and sulphur-oxidizers and probably play a key role in enriching these soils with bioavailable nitrate and sulfate [129–131]. Other organisms potentially contributing to inputs of bioavailable elements (e.g., organic carbon) to Antarctic soils are the photoautotrophic genus *Rhodoferax* [132]. All of these genera showed preferences for warmer habitats and probably have higher metabolic efficiencies at higher temperatures [133, 134]. Only *Conexibacter* showed a preference for lower temperature habitats. This genus has, in fact, been reported as widely present and dominant in Antarctic soils [135–137]. On the contrary, known cold-active taxa such as *Polaromonas* and *Aequorivita* showed a preference for higher temperature habitats (Fig. 7) [138, 139]. Acidophilic and acidotolerant genera (*Acidibacter* and *Bryobacter*) also showed preferences for habitats with warmer air temperatures: these genera were more abundant in vegetated sub-Antarctic island soils, which also typically exhibit lower pH values [140–142]. Taxa known to be extremely competitive in microbial communities, such as members of the genus *Lysobacter,* also showed higher temperature habitat preferences [143]. We suggest that taxa that exhibit habitat preferences have undergone some degree of habitat filtering [144] relating to the inherent climate conditions. It is therefore reasonable to hypothesize that these taxa are more likely to respond, either positively or negatively, to changes in climate than those taxa with more homogeneous distributions. Given that these taxa are dominant members of the community, it is likely that climate change-induced changes in their population numbers and/or function will impact the structure and function of the entire communities, and potentially also the soil geochemistry. Furthermore, all taxa that showed climatic preferences were also statistically identified as indicator genera of different ACBRs by LEfSe, showing that altered climatic conditions could also significantly affect bacterial communities, particularly the dominant taxa associated with different ACBRs.

Our study reports a comprehensive dataset from published and de novo Antarctic soil bacterial community datasets, where the combination of datasets from different sources has provided a viable means of circumventing the limitations imposed by the remoteness and sampling challenges of the Antarctic region. Despite the use of different 16S rRNA hyper-variable regions in generating the separate datasets used in this study, we argue that this is a valid approach, supported by the recently published study of Varliero et al., in which a subset of Antarctic soil samples was amplified with multiple 16S primer sets [91]. Analysis of the sequence data obtained indicated that the principal similarity/dissimilarity trends in bacterial community composition were effectively preserved, irrespective of the hyper-variable region amplified [91].

## Conclusions

We conclude that Antarctic soil bacterial diversity patterns and community structure identified in this study do not conform closely to the current ACBR classification, which is based primarily on data representing eukaryotic organisms, often from limited taxonomic groups. This might be due to the fact that prokaryotic soil communities, even in a single sample, are hugely diverse, represent a wide range of physiologies and functions, and are likely to be influenced by different distributional and dispersal drivers compared to eukaryotes. Soil prokaryotes may also exhibit higher levels of resistance and resilience to environmental stressors than higher organisms, which dominate the ACBR categories [110, 114, 118]. Furthermore, bacterial distributions are also based on soil heterogeneity and the presence of favorable microenvironments [85, 110, 114, 118]. Our results further suggest that Antarctic bacterial communities, and particularly the identified indicator taxa, might be impacted by climatic and other environmental changes in the different ACBRs [145].

The combined dataset used in this study represents a comprehensive baseline upon which future studies of Antarctic microbial ecology can be developed, and the outcomes of this study can also be used to bolster biodiversity conservation efforts on the continent and its associated islands. However, considering how soil geochemistry can vary at the micro-scale [146, 147] and how these microbial microenvironments and refugia are important in shaping microbial communities [118], future increased coverage of soil sampling design across Antarctica would allow for the development of a more robust ACBR-style classification including information derived from prokaryotic communities and also extending to islands that lie beyond the region of Antarctic Treaty governance. Therefore, further studies are clearly required and we emphasize the need for more extensive campaigns to systematically sample and better characterize Antarctic soil microbial communities. In particular, more even and representative mapping of Antarctic microbial distributions is required with, at present, some areas being relatively well studied (e.g., the Antarctic Peninsula and parts of Victoria Land) and others being heavily underrepresented [7, 139, 148]. Similarly, extensive microbial diversity and community characterization should also be extended to other microbial habitats such as rocks, freshwater lakes, and ice sheet environments [15, 149–151].

### Supplementary Information

**Additional file 1: Figure S1.** Analysed islands part of the Antarctic Conservation Biogeographic Regions (ACBRs). Geographic positions of islands included in the ACBR classification proposed by Terauds and Lee (2016) for ACBR1 and ACBR3 (A), ACBR 4 (B), ACBR 12 (C), ACBR 9 (D), ACBR 7 and ACBR16 (E-F), and ACBR 6 (G). **Figure S2.** Antarctic Conservation Biogeographic Regions (ACBR) unclassified islands (AUI). Sample locations are indicated by white dots. **Figure S3.** Bacterial Shannon diversity trends across ACBRs. Shannon diversity calculated using the genus dataset (A). Significant Tukey's statistical tests (*p* < 0.01) for Shannon diversity calculated at the genus-level (B). White dots correspond to non-significant Tukey’s statistical tests (*p* ≥ 0.01). ACBRs from 1 to 16 correspond to those described from Terauds and Lee (2016). ACBR 1: North-east Antarctic Peninsula; ACBR 3: North-west Antarctic Peninsula; ACBR 4: Central South Antarctic Peninsula; ACBR 6: Dronning Maud Land; ACBR 7: East Antarctica; ACBR 8: North Victoria Land; ACBR 9: South Victoria Land; ACBR 10: Transantarctic Mountains; ACBR 12: Marie Byrd Land; ACBR 16: Prince Charles Mountains. AUI: ACBR unclassified islands. **Figure S4.** Correlations between number of genera and bioclimatic variables. Pearson’s correlations between number of genera (i.e., richness) and BIO1 (A), BIO2 (B), BIO4 (C), BIO5 (D), BIO10 (E), BIO12 (F), BIO14 (G), BIO15 (H), BIO17 (I), BIO18 (J), SWE (K), distance to ocean (L) and elevation (M). BIO1: mean annual air temperature, °C; BIO2: mean diurnal air temperature range, °C; BIO4: temperature seasonality,°C/100; BIO5: mean daily maximum air temperature of the warmest month, °C; BIO10: mean daily mean air temperatures of the warmest quarter, °C; BIO12: annual precipitation, kg m^-2^; BIO14: precipitation in the driest month, kg m^-2^; BIO15: precipitation seasonality, %; BIO17: mean monthly precipitation in the driest quarter, kg m^-2^; BIO18: mean monthly precipitation in the warmest quarter, kg m^-2^; SWE: snow water equivalent, kg m^-2^; Distance to ocean: km; Elevation: m. **Figure S5.** Correlations between Shannon diversity and bioclimatic variables. Pearson’s correlations between Shannon diversity and BIO1 (A), BIO2 (B), BIO4 (C), BIO5 (D), BIO10 (E), BIO12 (F), BIO14 (G), BIO15 (H), BIO17 (I), BIO18 (J), SWE (K), distance to ocean (L) and elevation (M). BIO1: mean annual air temperature, °C; BIO2: mean diurnal air temperature range, °C; BIO4: temperature seasonality, °C/100; BIO5: mean daily maximum air temperature of the warmest month, °C; BIO10: mean daily mean air temperatures of the warmest quarter, °C; BIO12: annual precipitation, kg m^-2^; BIO14: precipitation in the driest month, kg m^-2^; BIO15: precipitation seasonality, %; BIO17: mean monthly precipitation in the driest quarter, kg m^-2^; BIO18: mean monthly precipitation in the warmest quarter, kg m^-2^; SWE: snow water equivalent, kg m^-2^; Distance to ocean: km; Elevation: m. **Figure S6.** Bioclimatic variables. Selected bioclimatic variables and characteristics for each ACBR and for ACBR unclassified islands (AUI): BIO1 (mean annual air temperature) (A), BIO2 (mean diurnal air temperature range) (B), BIO4 (temperature seasonality) (C), BIO10 (mean daily mean air temperatures of the warmest quarter) (D), BIO12 (annual precipitation amount) (E), BIO15 (precipitation seasonality) (F), BIO18 (mean monthly precipitation amount of the warmest quarter) (G), SWE (snow water equivalent) (H), elevation (I), distance from coast (J) and distance from ocean (K). All bioregions were reported except from H-J where AUI was excluded by data representation. ACBR 1: North-east Antarctic Peninsula; ACBR 3: North-west Antarctic Peninsula; ACBR 4: Central South Antarctic Peninsula; ACBR 6: Dronning Maud Land; ACBR 7: East Antarctica; ACBR 8: North Victoria Land; ACBR 9: South Victoria Land; ACBR 10: Transantarctic Mountains; ACBR 12: Marie Byrd Land; ACBR 16: Prince Charles Mountains. AUI: ACBR unclassified islands. **Figure S7.** Correlations between bacterial community composition and geographic distance, bioclimatic data, elevation, distance to coast and ocean. Relation between Bray-Curtis dissimilarity matrix performed on genus dataset and Euclidean distance matrix calculated for the entire dataset on geographic sample location (A) and on bioclimatic data (B), for the island dataset on geographic sample location (C) and on bioclimatic data (D), for the mainland dataset on geographic sample location (E), on bioclimatic data (F), on elevation (G), on distance to coast (H) and on distance to ocean (I). Bioclimatic data: BIO1, BIO2, BIO4, BIO5, BIO10, BIO12, BIO14, BIO15, BIO17, BIO18 and SWE associated to each sample. **Figure S8.** Variation partitioning performed for entire dataset and single ACBRs. Variation partitioning analyses performed on geography (distance) and bioclimatic variables for the entire dataset (A), AUI (B), ACBR 3 (C), ACBR 6 (D), ACBR 7 (E), ACBR 8 (F), ACBR 9 (G), ACBR 10 (H), ACBR 16 (I), only island samples (J), and only mainland samples (K). In addition to geography (distance) and bioclimatic variable, elevation and distances from coast and ocean were taken in consideration for variation partitioning performed only on mainland samples. ACBR 1: North-east Antarctic Peninsula; ACBR 3: North-west Antarctic Peninsula; ACBR 4: Central South Antarctic Peninsula; ACBR 6: Dronning Maud Land; ACBR 7: East Antarctica; ACBR 8: North Victoria Land; ACBR 9: South Victoria Land; ACBR 10: Transantarctic Mountains; ACBR 12: Marie Byrd Land; ACBR 16: Prince Charles Mountains. AUI: ACBR unclassified islands. **Figure S9.** Sample clustering at bioclimatic, bacterial community and geographic level. Tanglegram performed between dendrograms created using geography and bacterial community datasets (A) and bioclimatic and bacterial community datasets (B). Geography: geographical distances between samples in the form of latitude and longitude information; Bacterial community: Hellinger-transformed community at genus-level; Bioclimatic data: BIO1, BIO2, BIO4, BIO5, BIO10, BIO12, BIO14, BIO15, BIO17, BIO18 and SWE associated to each sample. ACBR 1: North-east Antarctic Peninsula; ACBR 3: North-west Antarctic Peninsula; ACBR 4: Central South Antarctic Peninsula; ACBR 6: Dronning Maud Land; ACBR 7: East Antarctica; ACBR 8: North Victoria Land; ACBR 9: South Victoria Land; ACBR 10: Transantarctic Mountains; ACBR 12: Marie Byrd Land; ACBR 16: Prince Charles Mountains. AUI: ACBR unclassified islands. **Figure S10.** ACBR 7 bacterial composition. PCoA  where only samples collected from ACBR 7 were collected and are colored in blue if from Vestfold hill region, and in red if from Windmill island region. **Figure S11.** dbRDA performed on only island samples or mainland samples. Distance-based redundancy analysis (dbRDA) performed on Hellinger transformed genus dataset and standardized bioclimatic variable dataset for only island samples (*n* = 142) (A) and only mainland samples (*n* = 846) (B). BIO2: mean diurnal air temperature range; BIO4: temperature seasonality; BIO10: mean daily mean air temperatures of the warmest quarter; BIO15: precipitation seasonality; BIO18: mean monthly precipitation amount of the warmest quarter; SWE: Snow water equivalent. **Figure S12.** Predictors of the dominant community distribution across Antarctica. Mean decrease accuracy associated to each bioclimatic variable (A). Number of taxa associated to the best predictor for each taxon distribution (predictor with highest %lncMSE) related to random forest analysis (B). BIO2: mean diurnal air temperature range; BIO4: temperature seasonality; BIO10: mean daily mean air temperatures of the warmest quarter; BIO15: precipitation seasonality; BIO18: mean monthly precipitation amount of the warmest quarter; SWE: Snow water equivalent. **Figure S13.** Relative abundance of dominant genera that were not selected by random forest model (variance explained < 30%). Only samples sequenced with V3-V4 and V4 16S rRNA primers were used for this analysis to ensure the best taxonomic consistency between samples (Varliero et al., 2023). Dominant genera were defined as those with a relative abundance of > 1% in at least one sample that were present in at least 10% of samples. Correspondingly, this approach included samples from AUI and ACBRs 1, 3, 4, 8, 9, 10 and 12. BIO2: mean diurnal air temperature range; BIO4: temperature seasonality; BIO10: mean daily mean air temperatures of the warmest quarter; BIO15: precipitation seasonality; BIO18: mean monthly precipitation amount of the warmest quarter; SWE: Snow water equivalent.

**Additional file 2:** **Table S1.** Specifics for all analysed datasets. The total number of samples was 1164, whereas the number of samples passing all the quality steps was 988. *ACBRs from 1 to 16 correspond to those described from Terauds and Lee (2016), "AUI" stands for “ACBR unclassified islands” and represents islands associated with the Antarctic mainland not included in the ACBR classification, and sub- and peri-Antarctic islands.  **years correspond to Austral summers except from when specified otherwise. ***number of samples after a cutoff of 5000 reads per sample was applied. **Table S2.** Sample specifics. BIO1: mean annual air temperature, °C; BIO2: mean diurnal air temperature range, °C; BIO4: temperature seasonality, °C/100; BIO5: mean daily maximum air temperature of the warmest month, °C; BIO10: mean daily mean air temperatures of the warmest quarter, °C; BIO12: annual precipitation, kg m^-2^; BIO14: precipitation in the driest month, kg m^-2^; BIO15: precipitation seasonality, %; BIO17: mean monthly precipitation in the driest quarter, kg m^-2^; BIO18: mean monthly precipitation in the warmest quarter, kg m^-2^; SWE: snow water equivalent, kg m^-2^; Distance to coast: km; Distance to ocean: km; Elevation: m. ACBRs from 1 to 16 correspond to those described from Terauds and Lee (2016). ACBR 1: North-east Antarctic Peninsula; ACBR 3: North-west Antarctic Peninsula; ACBR 4: Central South Antarctic Peninsula; ACBR 6: Dronning Maud Land; ACBR 7: East Antarctica; ACBR 8: North Victoria Land; ACBR 9: South Victoria Land; ACBR 10: Transantarctic Mountains; ACBR 12: Marie Byrd Land; ACBR 16: Prince Charles Mountains. AUI: ACBR unclassified islands. **Table S3.** Paramenters used in the dada2 function filterAndTrim() in each dataset. All the other options were set to default except for truncQ which was set to 0. **Table S4.** Number of reads at each step of the 16S rRNA gene processing pipeline for all datasets. *counts reported as read pairs. **Table S5.** Taxonomic relative abundance at phylum- (A), class- (B), order- (C) and family-level (D). **Table S6.** Relative abundance and taxonomy associated to genus dataset. **Table S7.** Analyses of variance (ANOVA) performed on bioclimatic variables, elevation and sample distance from coast/ocean. BIO1 (mean annual air temperature, °C), BIO2 (mean diurnal air temperature range, °C), BIO4 (temperature seasonality,°C), BIO5 (mean daily maximum air temperature of the warmest month, °C), BIO10 (mean daily mean air temperatures of the warmest quarter, °C), BIO12 (annual precipitation amount, kg m^-2^), BIO14 (precipitation amount of the driest month, kg m^-2^), BIO15 (precipitation seasonality, kg m^-2^), BIO17 (mean monthly precipitation amount of the driest quarter, kg m^-2^), BIO18 (mean monthly precipitation amount of the warmest quarter, kg m^-2^) and SWE (snow water equivalent, kg m^-2^). **Table S8.** Statistics from dbRDA (A-B) and variation partitioning (C-G). A and B only performed on bioclimatc varaibles selected by interactive dbRDA selection. Statistics from function varpart() with X1 as bioclimac dataset and X2 as geography (C) and individual statistical tests using anova.cca(): geography without controlling for environmental variables (D), environmental variables without controlling geography (E, geography alone (F) and environmental variables alone (G). **Table S9.** Indicator taxa across ACBRs and AUI at genus-level (LEfSe analysis based on Kruskal–Wallis *p* < 0.01).

## Data Availability

The datasets generated and/or analyzed during the current study are available in the National Center for Biotechnology Information (NCBI) repository under the accession PRJNA699250, PRJNA504513, PRJEB55870, PRJNA630822, PRJEB29853, PRJNA868884, PRJNA781801, PRJNA765625, PRJEB63224, PRJEB55309, PRJNA678471, PRJEB11689, PRJNA684555, PRJNA721735, PRJNA359740, and SRP067111 and also at https://data.bioplatforms.com/organization/australian-microbiome. The R scripts used for the analysis of the sequencing data can be found on the GitHub page https://github.com/gvMicroarctic/AntarcticBiogeographyPaper.
