# Supplementary material for: Biogeographic survey of soil bacterial communities across Antarctica
Source: Microbiome. 2024 Jan 12;12:9. doi: 10.1186/s40168-023-01719-3 (PMC10785390; doi:10.1186/s40168-023-01719-3)
Supplement: Supplementary file 2 — Additional file 1: Figure S1. Analysed islands part of the Antarctic Conservation Biogeographic Regions (ACBRs). Geographic positions of islands included in the ACBR classification proposed by Terauds and Lee (2016) for ACBR1 and ACBR3 (A), ACBR 4 (B), ACBR 12 (C), ACBR 9 (D), ACBR 7 and ACBR16 (E-F), and ACBR 6 (G). Figure S2. Antarctic Conservation Biogeographic Regions (ACBR) unclassified islands (AUI). Sample locations are indicated by white dots. Figure S3. Bacterial Shannon diversity trends across ACBRs. Shannon diversity calculated using the genus dataset (A). Significant Tukey's statistical tests (p < 0.01) for Shannon diversity calculated at the genus-level (B). White dots correspond to non-significant Tukey’s statistical tests (p ≥ 0.01). ACBRs from 1 to 16 correspond to those described from Terauds and Lee (2016). ACBR 1: North-east Antarctic Peninsula; ACBR 3: North-west Antarctic Peninsula; ACBR 4: Central South Antarctic Peninsula; ACBR 6: Dronning Maud Land; ACBR 7: East Antarctica; ACBR 8: North Victoria Land; ACBR 9: South Victoria Land; ACBR 10: Transantarctic Mountains; ACBR 12: Marie Byrd Land; ACBR 16: Prince Charles Mountains. AUI: ACBR unclassified islands. Figure S4. Correlations between number of genera and bioclimatic variables. Pearson’s correlations between number of genera (i.e., richness) and BIO1 (A), BIO2 (B), BIO4 (C), BIO5 (D), BIO10 (E), BIO12 (F), BIO14 (G), BIO15 (H), BIO17 (I), BIO18 (J), SWE (K), distance to ocean (L) and elevation (M). BIO1: mean annual air temperature, °C; BIO2: mean diurnal air temperature range, °C; BIO4: temperature seasonality,°C/100; BIO5: mean daily maximum air temperature of the warmest month, °C; BIO10: mean daily mean air temperatures of the warmest quarter, °C; BIO12: annual precipitation, kg m-2; BIO14: precipitation in the driest month, kg m-2; BIO15: precipitation seasonality, %; BIO17: mean monthly precipitation in the driest quarter, kg m-2; BIO18: mean monthly precipitation in the warmest quar [file 40168_2023_1719_MOESM1_ESM.docx]

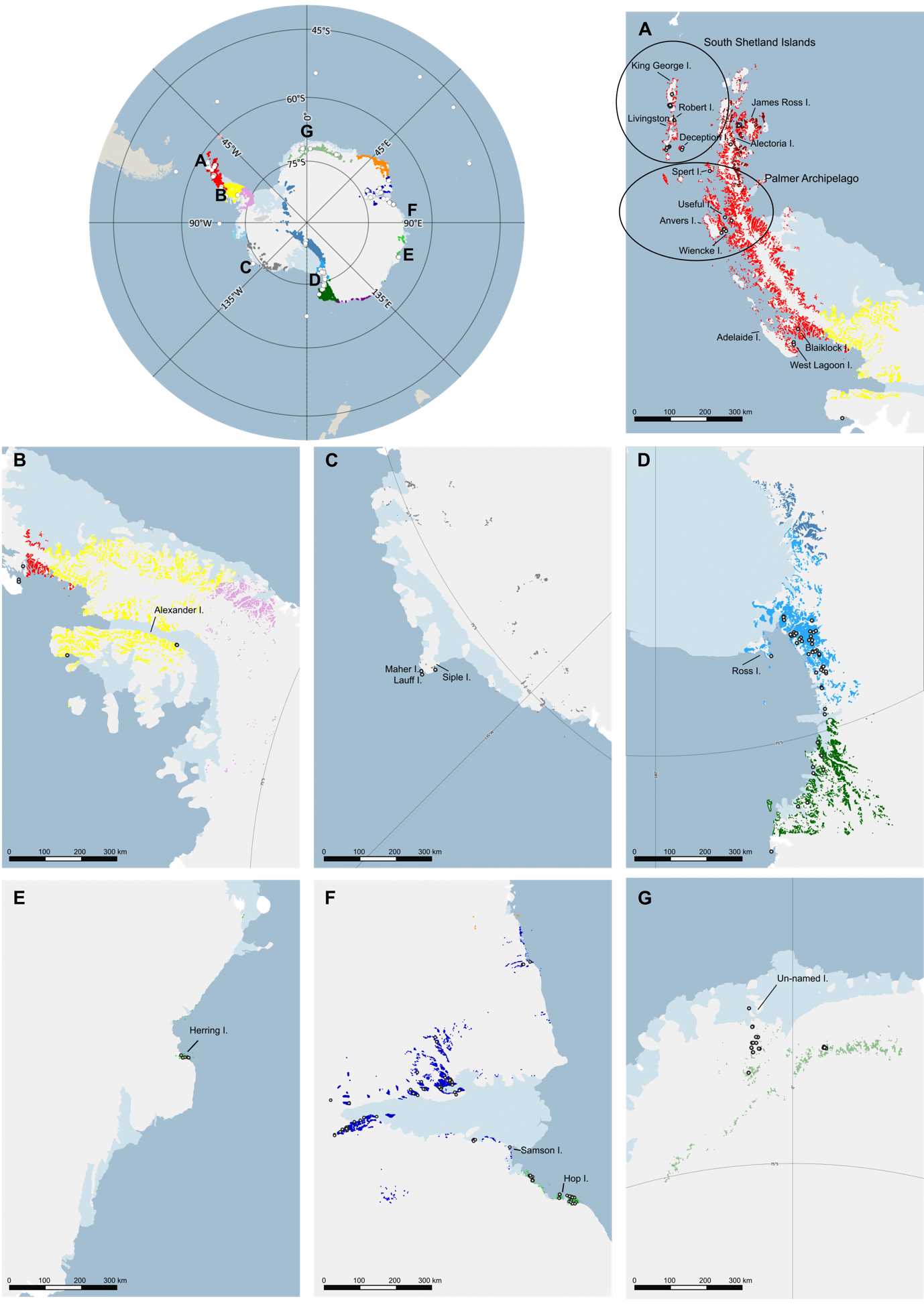
Figure S1. **Analysed islands part of the Antarctic Conservation Biogeographic Regions (ACBRs).** Geographic positions of islands included in the ACBR classification proposed by Terauds and Lee (2016) for ACBR1 and ACBR3 (A), ACBR 4 (B), ACBR 12 (C), ACBR 9 (D), ACBR 7 and ACBR16 (E-F), and ACBR 6 (G).


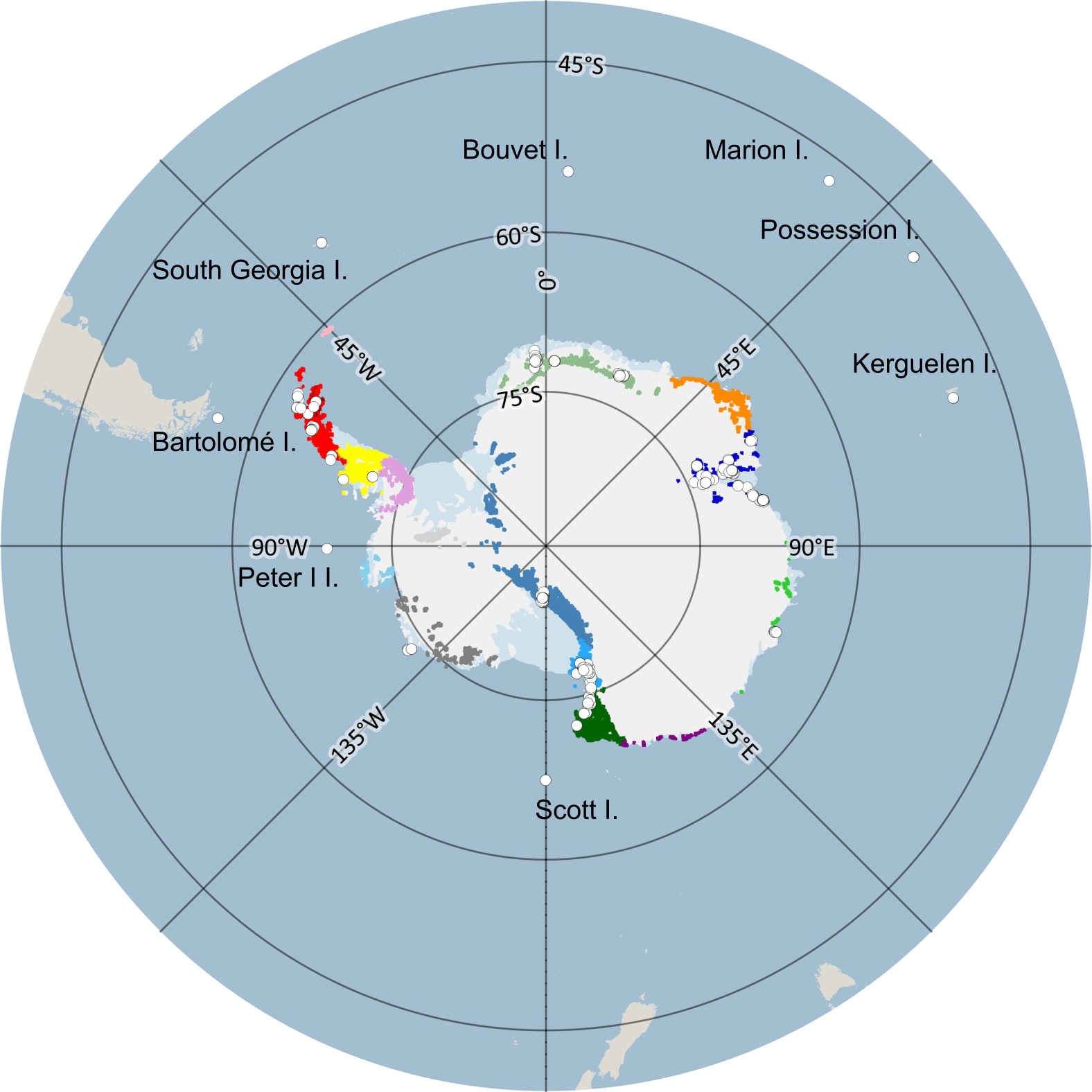


Figure S2. **Antarctic Conservation Biogeographic Regions (ACBR) unclassified islands (AUI).** Sample locations are indicated by white dots.


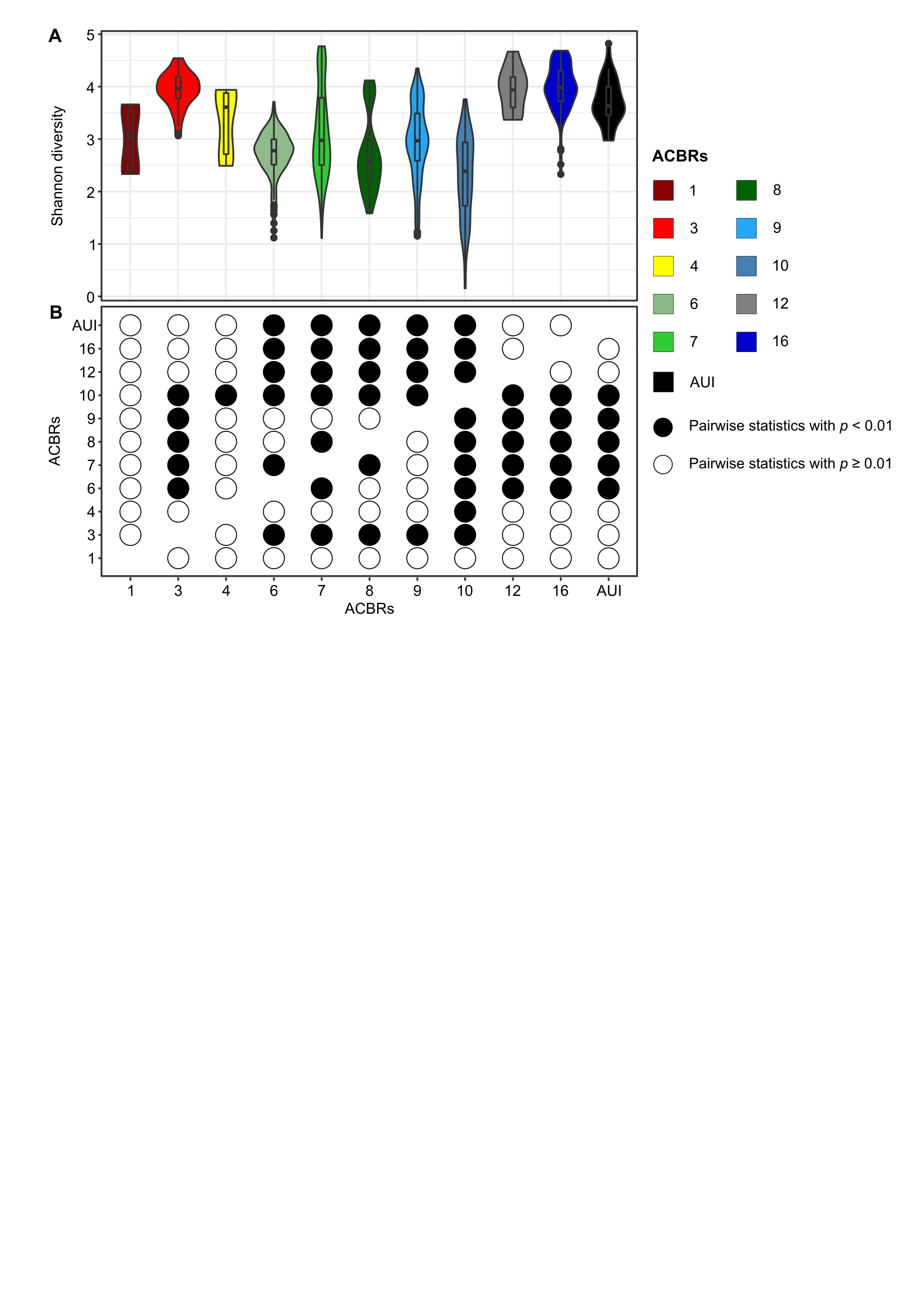


Figure S3. **Bacterial Shannon diversity trends across ACBRs.** Shannon diversity calculated using the genus dataset (A). Significant Tukey’s statistical tests (*p* < 0.01) for Shannon diversity calculated at the genus-level (B). White dots correspond to non-significant Tukey’s statistical tests (*p* ≥ 0.01). ACBRs from 1 to 16 correspond to those described from Terauds and Lee (2016). ACBR 1: North-east Antarctic Peninsula; ACBR 3: North-west Antarctic Peninsula; ACBR 4: Central South Antarctic Peninsula; ACBR 6: Dronning Maud Land; ACBR 7: East Antarctica; ACBR 8: North Victoria Land; ACBR 9: South Victoria Land; ACBR 10: Transantarctic Mountains; ACBR 12: Marie Byrd Land; ACBR 16: Prince Charles Mountains. AUI: ACBR unclassified islands.


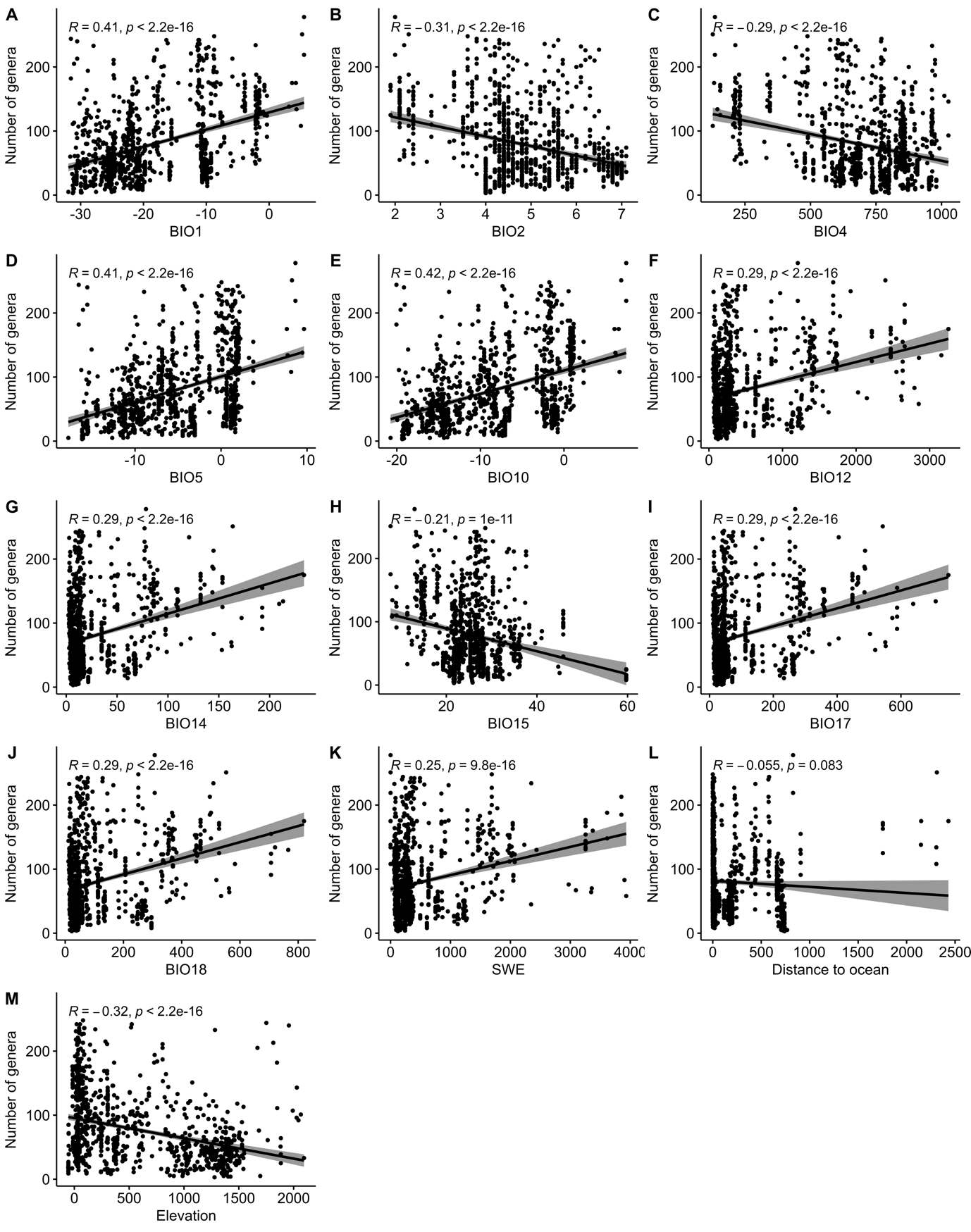


Figure S4. **Correlations between number of genera and bioclimatic variables.** Pearson’s correlations between number of genera (i.e., richness) and BIO1 (A), BIO2 (B), BIO4 (C), BIO5 (D), BIO10 (E), BIO12 (F), BIO14 (G), BIO15 (H), BIO17 (I), BIO18 (J), SWE (K), distance to ocean (L) and elevation (M). BIO1: mean annual air temperature, °C; BIO2: mean diurnal air temperature range, °C; BIO4: temperature seasonality, °C/100; BIO5: mean daily maximum air temperature of the warmest month, °C; BIO10: mean daily mean air temperatures of the warmest quarter, °C; BIO12: annual precipitation, kg m^-2^; BIO14: precipitation in the driest month, kg m-2; BIO15: precipitation seasonality, %; BIO17: mean monthly precipitation in the driest quarter, kg m^-2^; BIO18: mean monthly precipitation in the warmest quarter, kg m-2; SWE: snow water equivalent, kg m^-2^; Distance to ocean: km; Elevation: m.


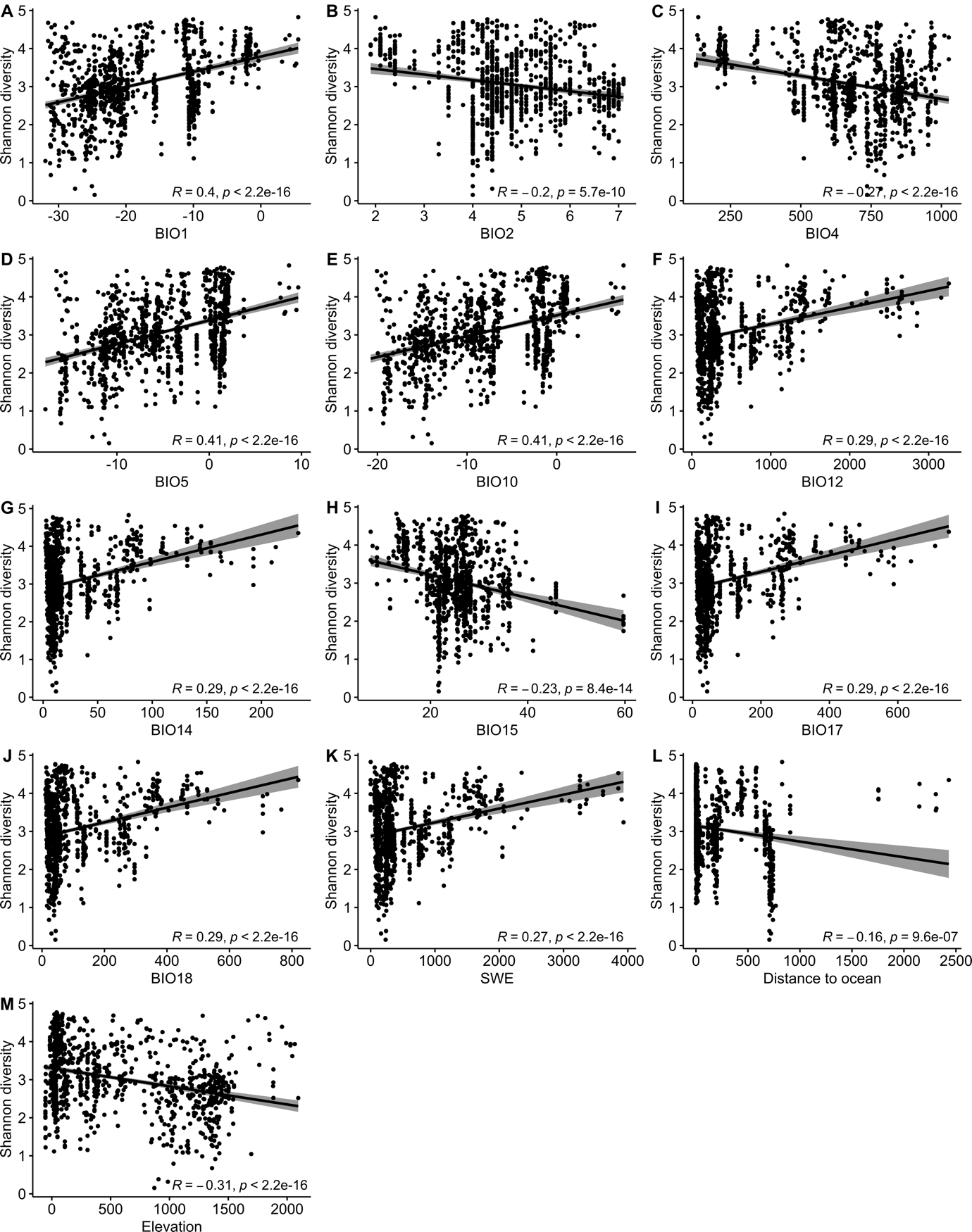


Figure S5. **Correlations between Shannon diversity and bioclimatic variables.** Pearson’s correlations between Shannon diversity and BIO1 (A), BIO2 (B), BIO4 (C), BIO5 (D), BIO10 (E), BIO12 (F), BIO14 (G), BIO15 (H), BIO17 (I), BIO18 (J), SWE (K), distance to ocean (L) and elevation (M). BIO1: mean annual air temperature, °C; BIO2: mean diurnal air temperature range, °C; BIO4: temperature seasonality, °C/100; BIO5: mean daily maximum air temperature of the warmest month, °C; BIO10: mean daily mean air temperatures of the warmest quarter, °C; BIO12: annual precipitation, kg m^-2^; BIO14: precipitation in the driest month, kg m-2; BIO15: precipitation seasonality, %; BIO17: mean monthly precipitation in the driest quarter, kg m^-2^; BIO18: mean monthly precipitation in the warmest quarter, kg m-2; SWE: snow water equivalent, kg m^-2^; Distance to ocean: km; Elevation: m.


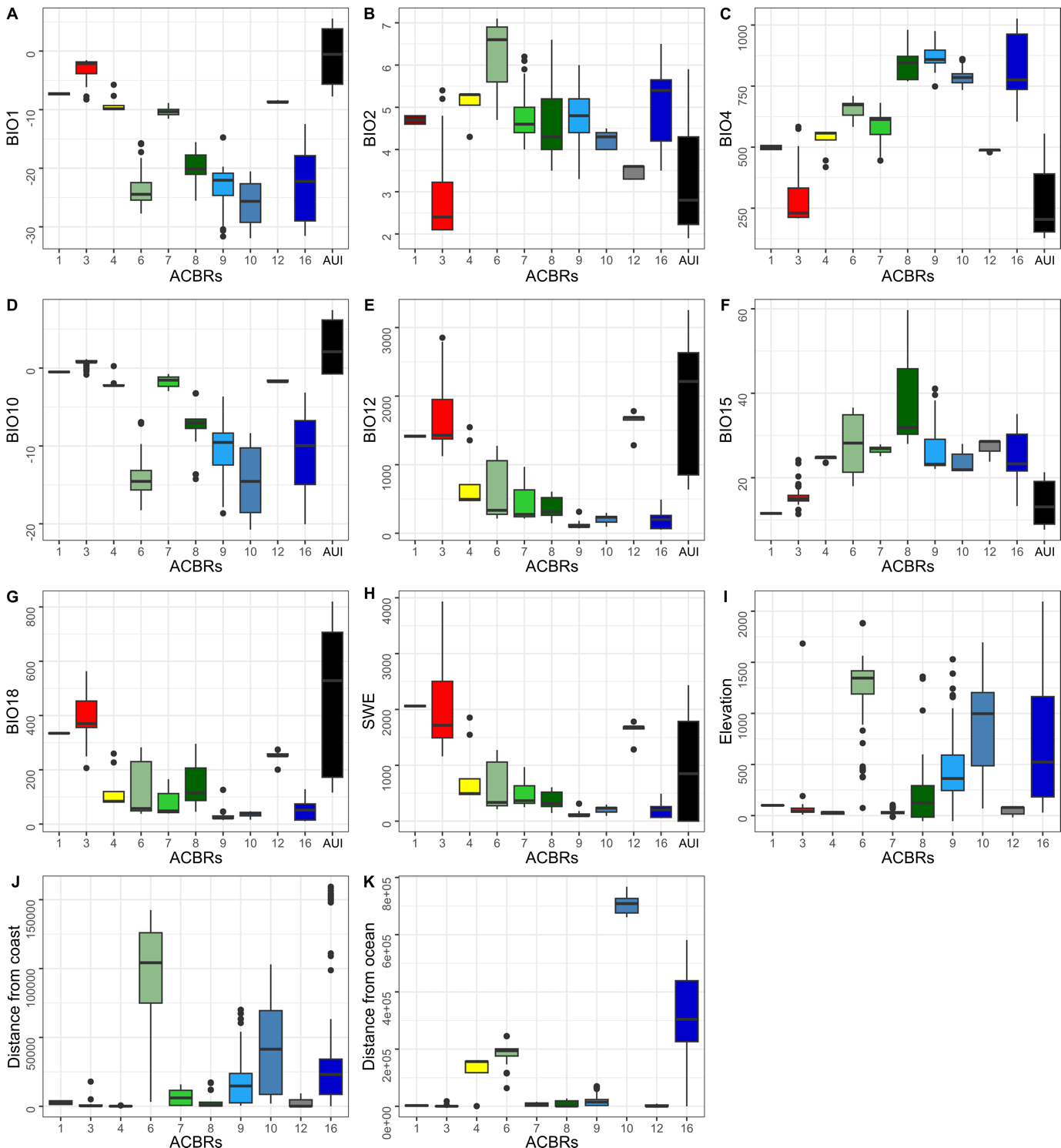


Figure S6. **Bioclimatic variables.** Selected bioclimatic variables and characteristics for each ACBR and for ACBR unclassified islands (AUI): BIO1 (mean annual air temperature) (A), BIO2 (mean diurnal air temperature range) (B), BIO4 (temperature seasonality) (C), BIO10 (mean daily mean air temperatures of the warmest quarter) (D), BIO12 (annual precipitation amount) (E), BIO15 (precipitation seasonality) (F), BIO18 (mean monthly precipitation amount of the warmest quarter) (G), SWE (snow water equivalent) (H), elevation (I), distance from coast (J) and distance from ocean (K). All bioregions were reported except from H-J where AUI was excluded by data representation. ACBR 1: North-east Antarctic Peninsula; ACBR 3: North-west Antarctic Peninsula; ACBR 4: Central South Antarctic Peninsula; ACBR 6: Dronning Maud Land; ACBR 7: East Antarctica; ACBR 8: North Victoria Land; ACBR 9: South Victoria Land; ACBR 10: Transantarctic Mountains; ACBR 12: Marie Byrd Land; ACBR 16: Prince Charles Mountains. AUI: ACBR unclassified islands.


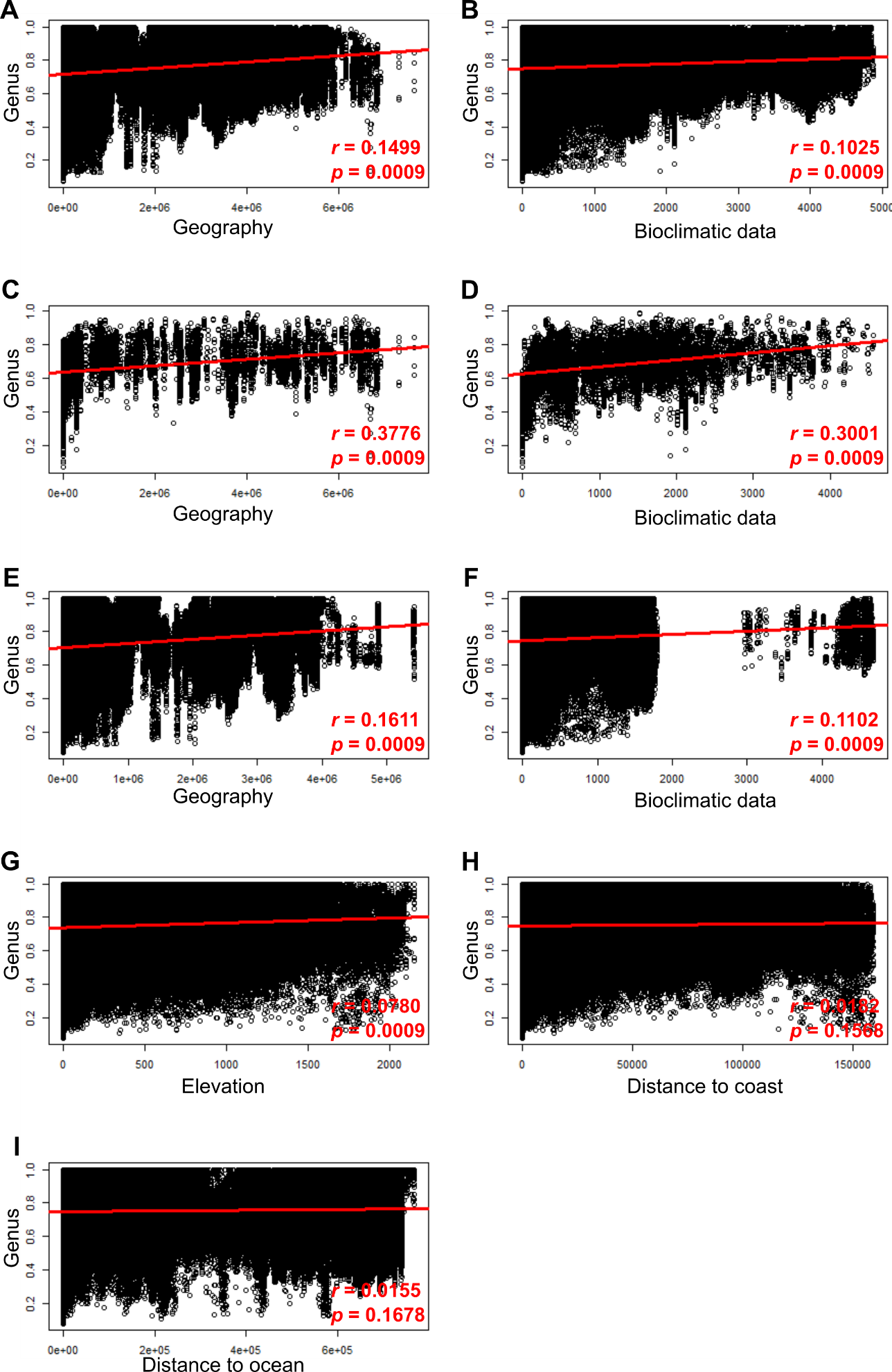


Figure S7. **Correlations between bacterial community composition and geographic distance, bioclimatic data, elevation, distance to coast and ocean.** Relation between Bray-Curtis dissimilarity matrix performed on genus dataset and Euclidean distance matrix calculated for the entire dataset on geographic sample location (A) and on bioclimatic data (B), for the island dataset on geographic sample location (C) and on bioclimatic data (D), for the mainland dataset on geographic sample location (E), on bioclimatic data (F), on elevation (G), on distance to coast (H) and on distance to ocean (I). Bioclimatic data: BIO1, BIO2, BIO4, BIO5, BIO10, BIO12, BIO14, BIO15, BIO17, BIO18 and SWE associated to each sample.


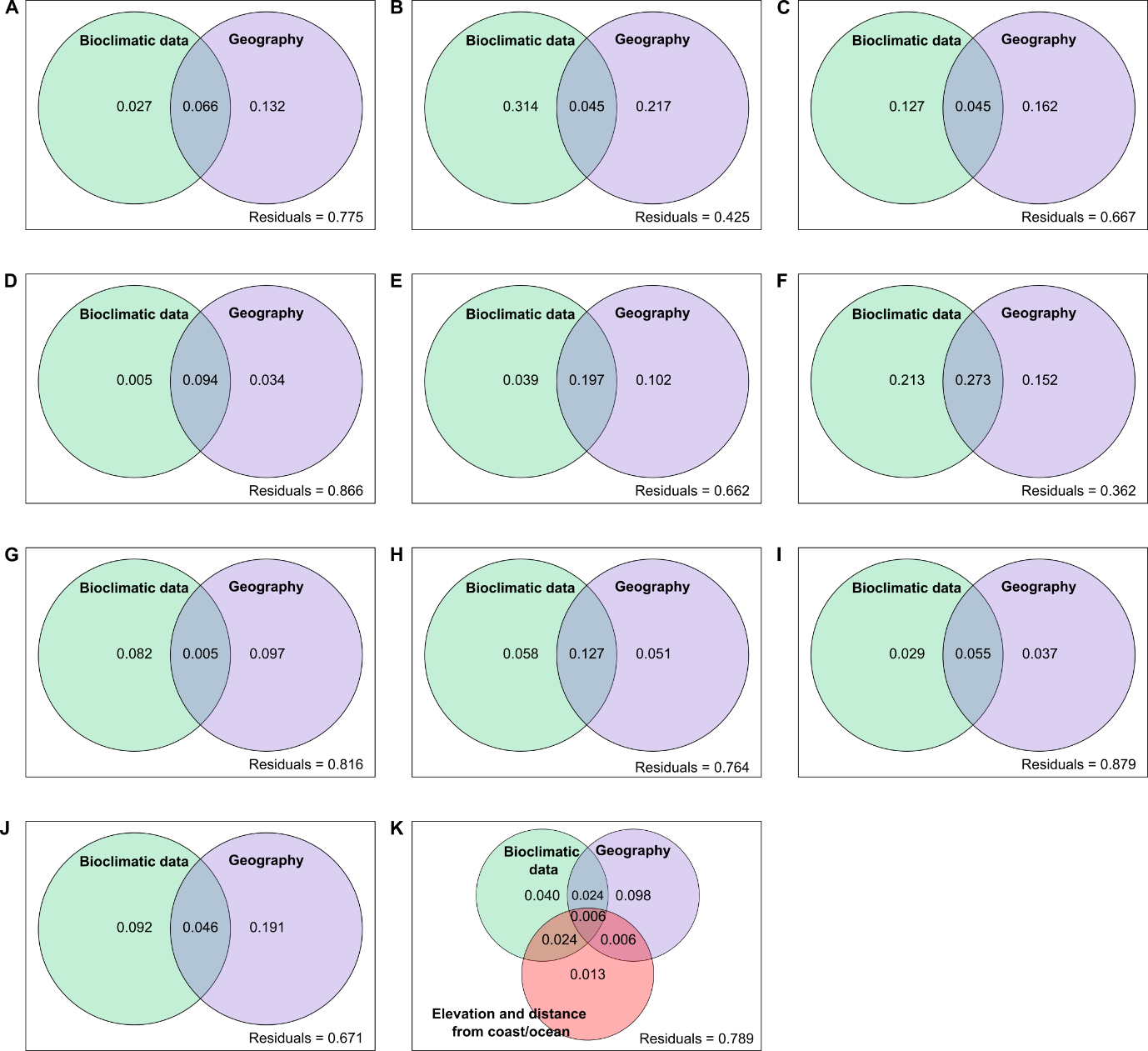


Figure S8. **Variation partitioning performed for entire dataset and single ACBRs**. Variation partitioning analyses performed on geography (distance) and bioclimatic variables for the entire dataset (A), AUI (B), ACBR 3 (C), ACBR 6 (D), ACBR 7 (E), ACBR 8 (F), ACBR 9 (G), ACBR 10 (H), ACBR 16 (I), only island samples (J), and only mainland samples (K). In addition to geography (distance) and bioclimatic variable, elevation and distances from coast and ocean were taken in consideration for variation partitioning performed only on mainland samples. ACBR 1: North-east Antarctic Peninsula; ACBR 3: North-west Antarctic Peninsula; ACBR 4: Central South Antarctic Peninsula; ACBR 6: Dronning Maud Land; ACBR 7: East Antarctica; ACBR 8: North Victoria Land; ACBR 9: South Victoria Land; ACBR 10: Transantarctic Mountains; ACBR 12: Marie Byrd Land; ACBR 16: Prince Charles Mountains. AUI: ACBR unclassified islands.


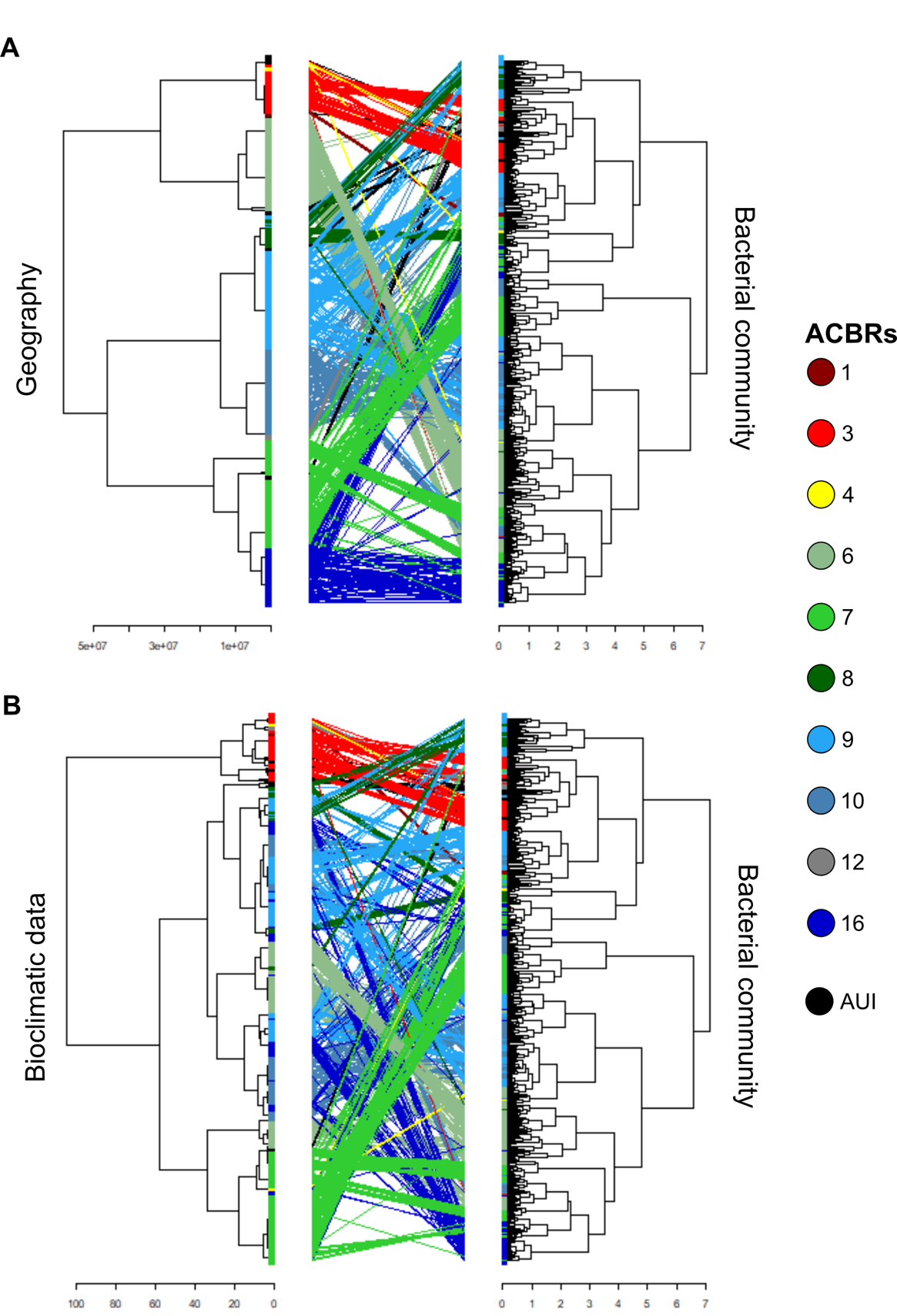


Figure S9. **Sample clustering at bioclimatic, bacterial community and geographic level.** Tanglegrams performed between dendrograms created using geography and bacterial community datasets (A) and bioclimatic and bacterial community datasets (B). Geography: geographical distances between samples in the form of latitude and longitude information; Bacterial community: Hellinger-transformed community at genus-level; Bioclimatic data: BIO1, BIO2, BIO4, BIO5, BIO10, BIO12, BIO14, BIO15, BIO17, BIO18 and SWE associated to each sample. ACBR 1: North-east Antarctic Peninsula; ACBR 3: North-west Antarctic Peninsula; ACBR 4: Central South Antarctic Peninsula; ACBR 6: Dronning Maud Land; ACBR 7: East Antarctica; ACBR 8: North Victoria Land; ACBR 9: South Victoria Land; ACBR 10: Transantarctic Mountains; ACBR 12: Marie Byrd Land; ACBR 16: Prince Charles Mountains. AUI: ACBR unclassified islands.


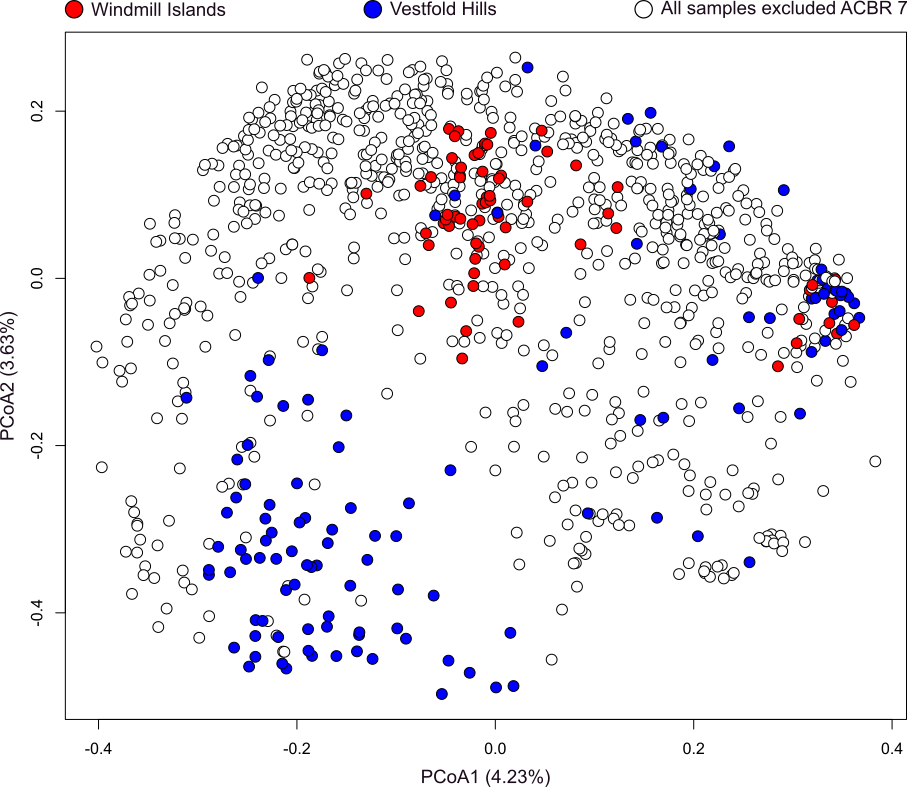


Figure S10. **ACBR 7 bacterial composition.** PCoA where only samples collected from ACBR 7 were collected and are colored in blue if from Vestfold hill region, and in red if from Windmill island region.

**
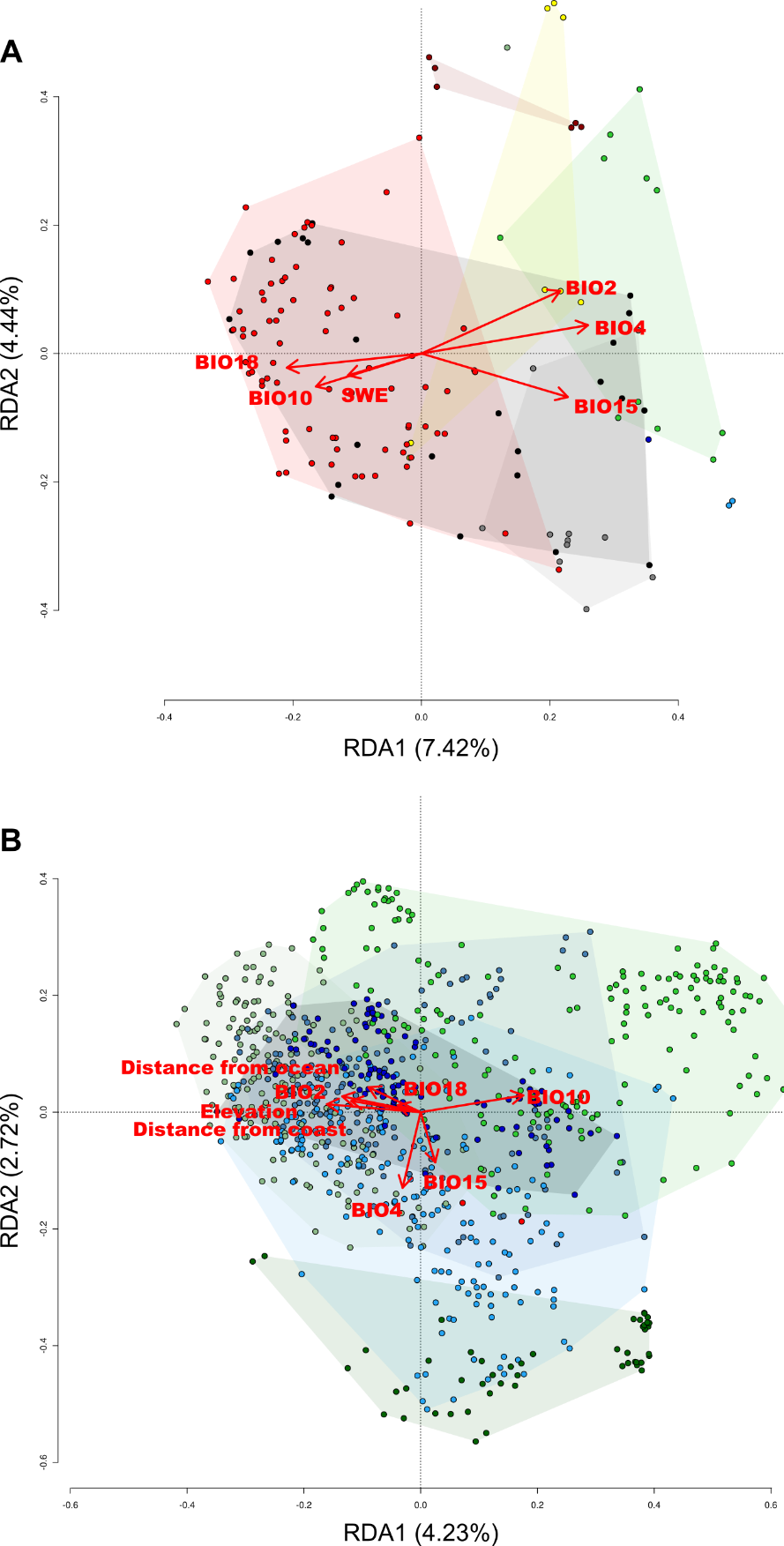
**

Figure S11. **dbRDA performed on only island samples or mainland samples.** Distance-based redundancy analysis (dbRDA) performed on Hellinger transformed genus dataset and standardized bioclimatic variable dataset for only island samples (*n* = 142) (A) and only mainland samples (*n* = 846) (B). BIO2: mean diurnal air temperature range; BIO4: temperature seasonality; BIO10: mean daily mean air temperatures of the warmest quarter; BIO15: precipitation seasonality; BIO18: mean monthly precipitation amount of the warmest quarter; SWE: Snow water equivalent.


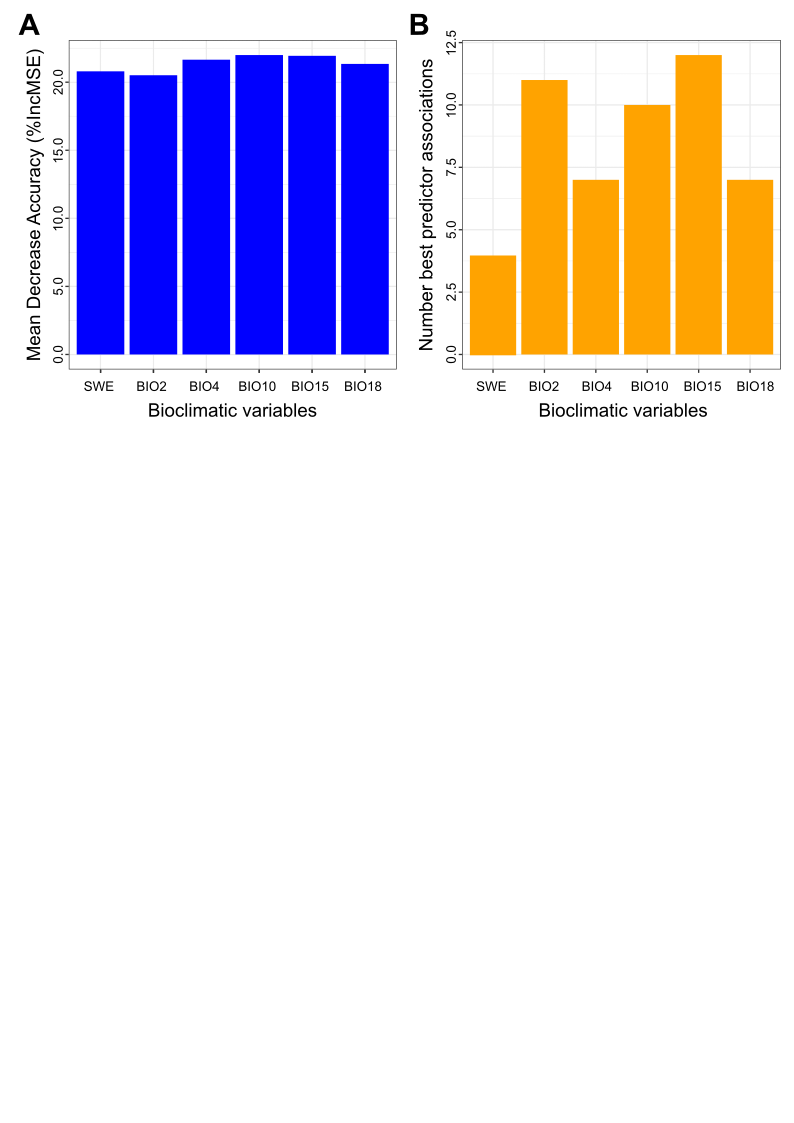
Figure S12. **Predictors of the dominant community distribution across Antarctica.** Mean decrease accuracy associated to each bioclimatic variable (A). Number of taxa associated to the best predictor for each taxon distribution (predictor with highest %lncMSE) related to random forest analysis (B). BIO2: mean diurnal air temperature range; BIO4: temperature seasonality; BIO10: mean daily mean air temperatures of the warmest quarter; BIO15: precipitation seasonality; BIO18: mean monthly precipitation amount of the warmest quarter; SWE: Snow water equivalent.


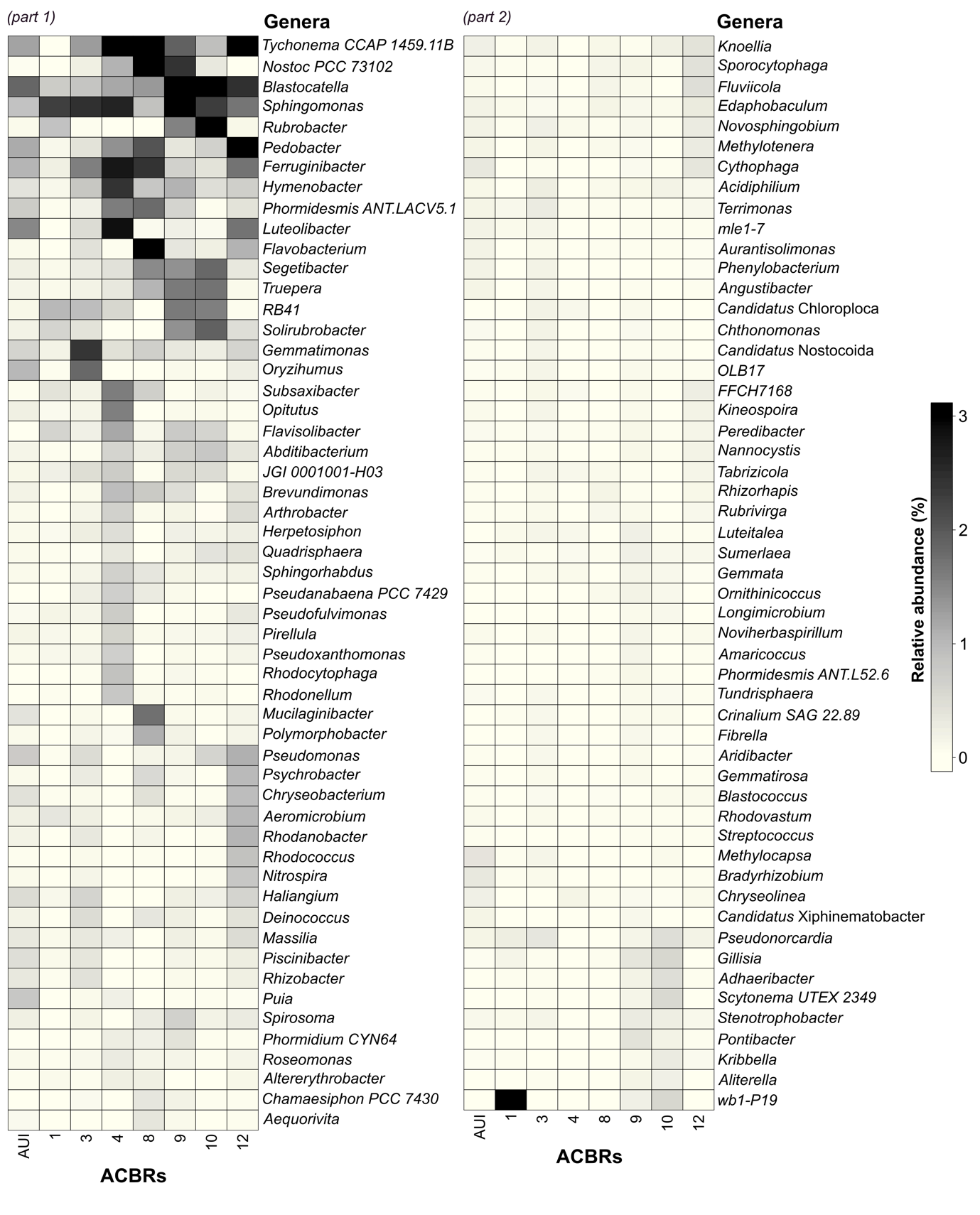
Figure S13. **Relative abundance of dominant genera that were not selected by random forest model (variance explained < 30%).** Only samples sequenced with V3-V4 and V4 16S rRNA primers were used for this analysis to ensure the best taxonomic consistency between samples (Varliero et al., 2023). Dominant genera were defined as those with a relative abundance of > 1% in at least one sample that were present in at least 10% of samples. Correspondingly, this approach included samples from AUI and ACBRs 1, 3, 4, 8, 9, 10 and 12. BIO2: mean diurnal air temperature range; BIO4: temperature seasonality; BIO10: mean daily mean air temperatures of the warmest quarter; BIO15: precipitation seasonality; BIO18: mean monthly precipitation amount of the warmest quarter; SWE: Snow water equivalent.
